# MicroRNA biogenesis is broadly disrupted by inhibition of the splicing factor SF3B1

**DOI:** 10.1093/nar/gkae505

**Published:** 2024-06-17

**Authors:** Angela Downie Ruiz Velasco, Aimee L Parsons, Matthew C Heatley, Athena R G Martin, Alfredo D Smart, Niraj Shah, Catherine L Jopling

**Affiliations:** School of Pharmacy, University of Nottingham, Nottingham NG7 2RD, UK; School of Pharmacy, University of Nottingham, Nottingham NG7 2RD, UK; The Digital Research Service, University of Nottingham, Nottingham, NG7 2RD, UK; School of Pharmacy, University of Nottingham, Nottingham NG7 2RD, UK; School of Pharmacy, University of Nottingham, Nottingham NG7 2RD, UK; The Digital Research Service, University of Nottingham, Nottingham, NG7 2RD, UK; School of Pharmacy, University of Nottingham, Nottingham NG7 2RD, UK

## Abstract

In animals, microRNA (miRNA) biogenesis begins with cotranscriptional cleavage of the primary (pri-)miRNA by the Microprocessor complex. Cotranscriptional splicing has been shown to influence Microprocessor cleavage when miRNAs are hosted in introns of protein-coding pri-miRNAs, but the impact of splicing on production of miRNAs hosted in long non-coding (lnc)RNAs is largely unknown. Here, we investigated the role of splicing in the biogenesis of miR-122, an lncRNA-hosted, highly expressed, medically important, liver-specific miRNA. We found that splicing inhibition by the SF3B1 inhibitor pladienolide B (PlaB) led to strong and rapid reduction in transcription of endogenous, but not plasmid-encoded, pri-miR-122, resulting in reduced production of mature miR-122. To allow detection of rapid changes in miRNA biogenesis despite the high stability of mature miRNAs, we used SLAMseq to globally quantify the effects of short-term splicing inhibition on miRNA synthesis. We observed an overall decrease in biogenesis of mature miRNAs following PlaB treatment. Surprisingly, miRNAs hosted in exons and introns were similarly affected. Together, this study provides new insights into the emerging role of splicing in transcription, demonstrating novel biological importance in promotion of miR-122 biogenesis from an lncRNA, and shows that SF3B1 is important for global miRNA biogenesis.

## Graphical Abstract

**Graphical Abstract:**
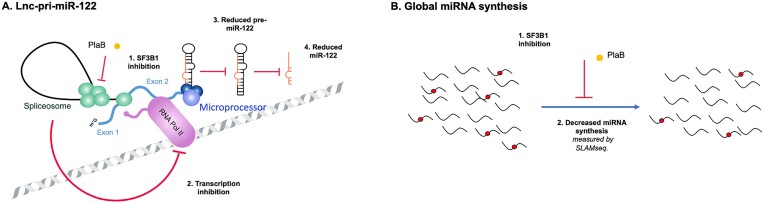

## Introduction

MicroRNAs (miRNAs) are 21–23-nucleotide (nt) non-coding RNA molecules expressed by a broad range of eukaryotic organisms. Metazoan miRNAs regulate gene expression by binding to partially complementary sites in target messenger RNAs (mRNAs), usually located in the 3′ untranslated region (UTR). This leads to reduced production of the encoded protein due to translation inhibition and/or mRNA degradation (1,2). In mammals, hundreds of individual miRNAs can each regulate a large number of targets, leading to complex combinatorial regulation that is important in normal development and is frequently dysregulated in disease, particularly cancer (1,3). Therapeutic strategies based on miRNA inhibition or overexpression thus hold considerable promise (3,4).

Mammalian miRNA biogenesis is a tightly coordinated, multistep process that results in tissue- and developmental stage-specific expression of different miRNAs (5,6). First, a precursor (pre-)miRNA hairpin is excised from a longer RNA polymerase II (RNAPII)-transcribed primary (pri-)miRNA by the Microprocessor complex, minimally composed of the endonuclease Drosha and double-stranded RNA-binding protein DGCR8. This is followed by nuclear export of the pre-miRNA and then cytoplasmic cleavage mediated by the endonuclease Dicer to generate an miRNA duplex, of which one strand is retained in the functional RNA-induced silencing complex (5,6). Extensive investigation has identified both sequence determinants of efficient miRNA biogenesis and protein factors that bind to, and influence processing of, specific miRNAs (7–11). While these studies have given considerable insights into factors determining cell-type-specific miRNA biogenesis, they have tended to study pre-miRNA hairpins outside their endogenous genomic context. However, in common with pre-mRNA processing events such as cleavage and polyadenylation (CPA), splicing and capping, Microprocessor cleavage occurs cotranscriptionally (12,13) and can therefore be influenced by the transcription process itself and by other cotranscriptional processing events. Genomic location of pre-miRNA hairpins is diverse, as they can be found in introns or exons of either protein-coding or long non-coding (lnc)RNA genes, either singly or as part of a cluster (1,5,6). In the latter case, a regulatory mechanism known as cluster assistance can promote biogenesis of suboptimal hairpins (14), illustrating the importance of genomic context for control of miRNA biogenesis.

Splicing in higher eukaryotes is tightly coupled to RNAPII transcription (15). Each intron removal event requires the stepwise assembly of the spliceosome, in which different small nuclear ribonucleoproteins (snRNPs) interact with the 5′ splice site (5′SS), branch point (BP) and 3′ splice site (3′SS) of an intron, leading to lariat formation, excision of the intron and ligation of the flanking exons (15). This is usually cotranscriptional, although post-transcriptional splicing also occurs to a variable extent (16,17). Early evidence in support of a regulatory link between miRNA biogenesis and splicing came from the observation that the Microprocessor and spliceosome interact (18). Most subsequent analyses of the relationship between splicing and miRNA processing have focused on approximately half of human miRNAs that are located in protein-coding pre-mRNAs, usually in introns (19–21). In this context, intronic miRNA cleavage was shown to precede splicing (12,18). Analysis of the relationship between miRNA biogenesis and splicing has shown different results in different studies, potentially due to gene-specific variation or differences in methodology. Splicing was shown to promote intronic miRNA biogenesis (22,23) or to slightly decrease it (24), while analysis of the effects of intronic miRNA processing on splicing of the host transcript showed promotion (23), inhibition (18) or no effect (12,24). While there are few examples of human miRNAs located in exons of protein-coding genes, a KSRP-modulated switch between the mutually exclusive production of exonic miR-198 and the spliced host *FSTL1* mRNA has been observed (25). Competition between splicing and miRNA production was also observed for a specialized category of miRNAs that overlap splice sites, designated splice overlapping (SO-)miRNAs, in minigene constructs (26,27). However, more recent work indicates that processing of endogenous SO-miRNAs does not interfere with splicing of neighbouring exons, emphasizing the importance of investigating miRNA processing in the endogenous genomic context (28).

In contrast, far less is known about the impact of splicing on production of ∼50% of human miRNAs that are hosted within lncRNAs (19–21). LncRNA is a broad definition applied to any RNA of >200 nt that does not encode a protein (29). LncRNAs are generally transcribed by RNAPII, which also transcribes pre-mRNAs. However, cotranscriptional processing, particularly splicing and CPA, is less efficient on lncRNAs than pre-mRNAs, and lncRNAs have fewer introns (16,30,31). Previously, we identified a novel mechanism of transcription termination in pri-miR-122, an lncRNA that hosts the highly expressed, liver-specific miR-122 (32,33). miR-122 is medically important as it is required for hepatitis C virus (HCV) replication and acts as a tumour suppressor in hepatocellular carcinoma (34,35). We found that Microprocessor cleavage at the pre-miR-122 hairpin terminates transcription directly, generating ∼4.8 kb (unspliced) and ∼1.9 kb (spliced) non-polyadenylated transcripts (33). Moreover, we found that while plasmid-encoded pri-miR-122 switches to CPA when Microprocessor cleavage is inhibited, the endogenous pri-miR-122 gene is not competent for transcription termination by CPA even in these circumstances (33). Genome-wide analysis demonstrated that Microprocessor-mediated transcription termination is also employed by a number of other lnc-pri-miRNAs, but not by protein-coding pri-miRNAs, demonstrating an important difference in the cotranscriptional processing of these two classes of pri-miRNA transcript (33).

Here, we investigated the interplay between splicing and Microprocessor cleavage on pri-miR-122 in order to understand how these cotranscriptional processing events interact in the context of an lncRNA and how high expression of mature miR-122 is achieved in the liver. We induced global inhibition of splicing in Huh7 human hepatocellular carcinoma cells, in which miR-122 is highly expressed, using the small molecule anti-tumour compound pladienolide B (PlaB), an inhibitor of the SF3B1 component of the U2 snRNP (36,37). PlaB caused a rapid reduction in transcription of pri-miR-122 in its endogenous context, but interestingly did not induce this effect when pri-miR-122 was expressed from a plasmid under control of a heterologous promoter in HeLa cells, emphasizing the importance of investigating miRNA biogenesis in the endogenous context. Short-term PlaB treatment did not affect total mature miR-122 levels, presumably due to high stability of the mature miRNA, but caused a strong decrease in pre-miR-122. To specifically analyse new miRNA synthesis, we employed the recently developed genome-wide approach of SLAMseq (38), based on 4-thiouridine (4SU) incorporation into nascent RNA and subsequent chemical conversion such that 4SU positions are identified by T>C transitions in RNA sequencing (RNA-seq). SLAMseq confirmed that PlaB inhibits miR-122 biogenesis and unexpectedly showed that it also leads to a global decrease in miRNA production. While synthesis of individual miRNAs was differentially affected by PlaB, we did not observe any influence of genomic context, or *cis*-elements that promote Microprocessor cleavage, on the response to PlaB. Finally, we found that direct inhibition of endogenous pri-miR-122 splicing by antisense oligonucleotide (ASO) transfection or CRISPR modification did not reproduce the transcriptional effects of PlaB, suggesting that the role of SF3B1 in transcription is distinct from its function in intron recognition and removal. Detailed analysis of the effect of splicing on pri-miR-122 processing independent of transcriptional regulation by PlaB showed a small positive effect on cotranscriptional Microprocessor cleavage, but overall our data suggest that SF3B1 mainly regulates miRNA biogenesis at the level of transcription. Together, our results provide new insights into the factors that control miRNA biogenesis and identify a previously unknown role of SF3B1 in promoting the accumulation of high levels of miR-122 by driving efficient transcription of the host lncRNA.

## Materials and methods

### Cell culture, PlaB treatment and transfection

Huh7 and HeLa cells were maintained in high-glucose Dulbecco’s modified Eagle medium (Sigma) supplemented with 10% fetal bovine serum (Gibco). For Huh7 culture, 1% non-essential amino acids (Invitrogen) were also included in the culture media. PlaB (Sigma) was dissolved in dimethyl sulfoxide (DMSO), stored in aliquots at −20°C and added to cells in fresh medium at a final concentration of 1 μM for 4 h, unless otherwise indicated. An equivalent volume of DMSO was used as a control. A plasmid encoding pri-miR-122 under control of the HIV LTR promoter has been previously described (33). Lipofectamine 2000 (Invitrogen) was used to deliver 0.1 μg pri-miR-122 plasmid and 0.025 μg pTAT per well of a six-well plate, 24 h before PlaB treatment. ASOs fully modified with 2′-methyoxyethyl and a phosphorothioate backbone were delivered to cells using Lipofectamine RNAiMax (Invitrogen). ASOs were delivered at 50 nM final concentration and cells were cultured for 24 h before harvesting total or chromatin-associated RNA. ASOs were custom designed and purchased from IDT, and sequences are shown in Supplementary Table S1.

### CRISPR/Cas9 modification of pri-miR-122

Cas9 RNP was formed by incubation of 150 pmol Cas9-NLS (Horizon) with 133 pmol each of two EDIT-modified custom single-guide RNAs (sgRNAs; Horizon). sgRNAs were designed to generate a deletion spanning the branch point, polypyrimidine tract and 3′SS of pri-miR-122 and targeted the sequences ACCTGTAAAGGTTGATTTGG and AGGATGCTCAGATACTGCTA, respectively. Cas9 RNP was delivered into Huh7 cells using a Nucleofector D (Lonza) in 100 μl buffer SE using the programme CM-104, as previously shown to be effective in Huh7.5 cells (39). Individual clonal cell lines were isolated by seeding in 96-well plates at single cell dilution and were screened by polymerase chain reaction (PCR) of genomic DNA using the primers 3′SS gDNA F and 3′SS gDNA R (Supplementary Table S2). Successful deletion of the targeted region in one allele was confirmed by Sanger sequencing of the wild-type (WT) and deletion PCR products for two clonal 3′SSΔ cell lines.

### RNA isolation

Total RNA was extracted from cells by direct addition of TRI Reagent (Sigma) to the plate. Isolation followed the manufacturer’s instructions, with the addition of TURBO DNase (Thermo Fisher) treatment when RNA was to be used for reverse transcription quantitative PCR (RT-qPCR). For miRNA northern blots, small RNA was isolated from the total RNA population using RNA Clean & Concentrator columns (Zymo) and following the manufacturer’s protocol for isolation of 17–200 nt RNA.

### Chromatin fractionation

Isolation of chromatin-associated RNA was performed as described previously (40), with addition of a final TURBO DNase (Thermo Fisher) treatment followed by isolation using RNA Clean & Concentrator columns (Zymo). Successful separation of the chromatin fraction was confirmed by western blot (Supplementary Figure S2A).

### Reverse transcription quantitative PCR (RT-qPCR)

Reverse transcription was carried out using GoScript (Promega) with random primers, with 100 ng total RNA extracted using TRI Reagent and treated with DNase as a template. One microlitre of the reverse transcription reaction was used as a template for qPCR using GoTaq (Promega). Primer sequences are shown in Supplementary Table S2. Reactions were carried out in a RotorGene (Qiagen) with standard cycling parameters. Following cycling, a melt curve programme was run to check for primer specificity. All qPCR reactions were carried out in technical triplicates, and no-RT controls were included to control for genomic or plasmid DNA contamination. RT-qPCR data were analysed using the 2^−ΔΔCt^ method relative to 18S ribosomal RNA (rRNA) or actin mRNA, or relative to input for RNA immunoprecipitation (RIP). Both housekeeping controls were confirmed to be unchanged by PlaB treatment in total RNA. For analysis of nascent RNA, 18S rRNA was used as the housekeeping control as it was possible that actin mRNA would be affected by PlaB at the nascent level. GAPDH pre-mRNA was used as a housekeeping control for chromatin-associated RNA in ASO experiments as global splicing was not disrupted.

RT-qPCR for mature miR-122 was carried out using TaqMan miRNA assays (Thermo Fisher) to hsa-miR-122 (assay ID 002245) and U6 snRNA (assay ID 001973) as a housekeeping control. miRNA RT-qPCR data were analysed using the 2^−ΔΔCt^ method.

### Northern blotting

Northern blotting for pri-miR-122 was carried out as described (33). Blots were stripped and re-probed for γ-actin mRNA as a loading control. Imaging was carried out using a Typhoon Phosphorimager (GE Healthcare). To carry out northern blotting for pre- and mature miRNAs, 55 μg of <200 nt RNA was separated by 15% polyacrylamide/7 M urea/0.5× TBE gel electrophoresis. ^32^P-labelled Decade ladder (Ambion) was included as a size marker. RNA was transferred to Hybond N+ membrane (Amersham) by semi-dry transfer in water for 1 h at 4°C. Membranes were UV cross-linked in a Stratalinker (Strategene) and pre-hybridized by rotation for 30 min at 37°C in a hybridization chamber in ULTRAhyb-Oligo (Ambion). Hybridization was carried out by overnight rotation at 37°C in fresh ULTRAhyb-Oligo supplemented with ^32^P end-labelled DNA oligonucleotide complementary to the miRNAs of interest or U6 snRNA (Supplementary Table S3). Membranes were washed according to the manufacturer’s instructions and imaged using a Storm Phosphorimager (GE Healthcare). Membranes were stripped with three successive 30 min incubations in boiling water with sodium dodecyl sulfate (SDS) added to 0.5%, and removal of signal confirmed by phosphorimaging before re-probing.

### RNA immunoprecipitation

The cross-linked RIP protocol was adapted from (41). Huh7 cells grown to 80–90% confluence in 10 cm plates ± PlaB treatment were cross-linked in 1% formaldehyde for 10 min at room temperature. The reaction was stopped by addition of 0.1 volume of 2.66 M glycine and incubation for 10 min at room temperature followed by 5 min on ice. Cells were collected by centrifugation and lysed in 1 M Tris–HCl (pH 7.4), 5 M NaCl and 0.5% NP-40 for 15 min on ice, followed by Dounce homogenization. Nuclei were recovered by centrifugation (1000 × *g*, 10 min, 4°C), resuspended in nuclei resuspension buffer [1 M HEPES–KOH, pH 7, 2 M MgCl_2_, 1 mM dithiothreitol (DTT), RNaseOUT, protease inhibitor] and sonicated for 2 × 5 cycles of 15 s on and 45 s off. Lysates were treated with TURBO DNase (Thermo Fisher) for 30 min at 37°C with shaking and centrifuged to remove debris. For conjugation, 50 μl of Dynabeads Protein A (Invitrogen) was washed twice and resuspended in nuclei resuspension buffer supplemented with 1% Triton X-100, 0.1% sodium deoxycholate, 0.01% SDS and 140 mM NaCl, and then rotated at room temperature for 1 h with 5 μg anti-DGCR8 antibody (Abcam ab191875) or a rabbit IgG isotype control (Invitrogen). Beads were then washed six times with complete nuclei resuspension buffer. Equal volumes of lysate were then added to the conjugated beads, with 1/10 volume set aside as input control, and rotated overnight at 4°C. Beads were washed five times in ice-cold high-salt wash buffer [1 M Tris–HCl, pH 7.4, 5 M NaCl, 0.5 M ethylenediaminetetraacetic acid (EDTA), 0.5% NP-40] and RNA eluted by incubation in Proteinase K buffer (50 mM Tris–HCl, pH 7.4, 150 mM NaCl, 1 mM MgCl_2_, 0.05% NP-40, 1% SDS, 1.2 mg/ml Proteinase K) for 30 min at 55°C with shaking. RNA was extracted from input and IP samples using TRIzol LS (Thermo Fisher).

### 4SU labelling and pulldown

Huh7 cells were grown to 70% confluence in 10 cm plates and treated with PlaB or DMSO for 4 h total, with 200 μM 4SU (Sigma; stored in aqueous solution in foil-wrapped aliquots at −20°C) added for the final 1 h of incubation. Under red light to minimize UV exposure, cells were washed twice in ice-cold phosphate-buffered saline (PBS) and total RNA extracted with TRI Reagent, heated to 65°C for 5 min and cooled immediately on ice. Subsequent biotinylation and purification of 4SU-labelled RNA was adapted from (42,43). Thirty-five micrograms of RNA was incubated with 5 μg MTSEA-biotin-XX (Biotium, 100 μg/ml stock in dimethylformamide), 10 mM HEPES (pH 7.5) and 1 mM EDTA in 250 μl total volume and rotated in the dark for 30 min at room temperature. RNA was purified by two 1:1 chloroform extractions, the second of which used a MaXtract column (Qiagen). RNA was precipitated with 0.1 volume of 5 M NaCl and 1 volume of isopropanol, washed in 70% ethanol, resuspended in 50 μl water, heated to 65°C for 5 min and cooled immediately on ice. Fifty microlitres of μMACS streptavidin beads (Miltenyi) were added and mixed by rotation at room temperature for 15 min. μMACS columns were equilibrated with wash buffer (100 mM Tris–HCl, pH 7.4, 10 mM EDTA, 1 M NaCl, 0.1% Tween 20) and then the RNA–streptavidin bead mix was allowed to flow through the columns. Columns were washed five times in wash buffer pre-heated to 65°C. RNA was eluted twice with 100 μl freshly prepared 100 mM DTT and precipitated with 0.1 volume of NaCl, 1 volume of isopropanol and 1 μl GlycoBlue (Ambion). RNA was resuspended in 20 μl water and equal volumes used for complementary DNA synthesis.

### Actinomycin D transcriptional block

Huh7 cells were grown in 10 cm plates. Cells were first incubated for 1 h with 4SU at a final concentration of 250 μM. After 1 h, cells were washed three times with PBS before replacing media and adding actinomycin D (Act D) at a final concentration of 5 μg/ml. After 15 min, either PlaB at a final concentration of 1 μM or an equivalent volume of DMSO was added and cells were incubated for a further 15 min before proceeding with biotinylation and pulldown.

### SLAMseq

Huh7 cells grown to 70% confluence in 10 cm plates were treated with 500 μM 4SU for 4 h, concurrent with either DMSO or PlaB treatment. Subsequent steps were carried out under red light to minimize UV exposure. Total RNA was isolated using TRI Reagent, and 8 μg was chemically modified as in (44). Four independent experiments were carried out, each with *Control* (no 4SU, DMSO), *DMSO* (4SU, DMSO) and *PlaB* (4SU, PlaB) conditions. RNA quality control (QC), library preparation and sequencing were carried out by Lexogen. RNA QC was carried out using a Fragment Analyzer (Agilent), with all RNA samples giving an RNA quality number of ≥9.9. Libraries were prepared using 600 ng RNA with the NEXTflex Small RNA-Seq Kit (PerkinElmer), according to the manufacturer’s protocol. Libraries were pooled before excision from a 3% agarose gel. Sequencing was carried out using the single read (SR100) mode on a NextSeq 2000 (Illumina). Two samples (DMSO A and DMSO B) produced a low number of reads on first sequencing. To address this, new libraries were prepared for these two samples, and existing libraries were also re-amplified. Both re-prepped and re-amplified libraries were sequenced, and reads from the three runs (original, re-prepped and re-amplified) were combined for analysis.

### Bioinformatic analysis

The SLAM-DUNK pipeline (45) that was previously developed for determination of T>C conversions in 3′UTRs analysed by QuantSeq was adapted to examine mature miRNA sequences. To this end, RNA-seq data were processed via the nf-core workflow SLAMseq v1.0.0 (https://nf-co.re/slamseq/1.0.0) (46) with GRCh38 (GCA 000001405.15) as the reference genome and DMSO (4SU, DMSO) set as the control for its DEseq2 analysis. However, coordinates for 3′UTR features were substituted with those of mature hsa miRNAs obtained from miRBase v22 (47) in order to report the relevant T>C conversion rates. To remove randomized adapters included in the library preparation and to ensure that only mature miRNA sequences were analysed, reads were trimmed by an additional 4 bp at the 3′ ends (--three prime clip R1 4) and then filtered to discard those longer than 30 bp (--max length 30) during adapter removal via Trim Galore!, with a further 4 bp trimmed from 5′ ends when mapped by SLAM-DUNK (--trim5 4). Alignment filtering by SLAM-DUNK was also altered to remove the mapping quality threshold (-mq 0) so that miRNAs mapping to >1 genomic location were retained. The filter for the number of mismatches allowed within a read was set to three (-nm 3), although the default mapping integrity filter (mi 0.95) overcomes this for miRNA reads. Single-nucleotide polymorphism (SNP) masking was also altered to reduce the minimum alternate allele frequency to 0.4, as this would allow detection of any SNPs at diploid loci (--var fraction 0.4). Otherwise, default SLAMseq settings were used throughout. Reads were analysed for any quality issues by FastQC.

Analysis was limited to miRNAs with expression >100 CPM (counts per million) in all datasets. To ensure that only high-confidence miRNAs were considered, miRNAs that were absent from the manually curated database MirGeneDB 2.1 (https://mirgenedb.org) (48) were also excluded. miRNAs fulfilling both these criteria form the ‘Initial miRNA Dataset’ used for initial characterization of the sequencing data and comparison between treatments and experiments. To confirm reproducibility between the four independent experiments, a mixed effects linear model was fit that showed no random effect from different experiments in either CPM or T>C conversion rates.

For quantification of the effects of PlaB on synthesis of specific miRNAs, the DEseq2 comparison of T>C conversion rate in *Control* and *DMSO* conditions generated by SLAM-DUNK was used to exclude miRNAs that were not labelled above background. Only miRNAs that were higher in *DMSO* than *Control* with *P*_adj_ > 0.05 were considered as significantly labelled and carried forward to further analysis. miRNAs were also excluded if the average T>C conversion rate in *PlaB* was below that of *Control*. The resulting ‘Final miRNA Dataset’ was used for all subsequent annotation and analysis (Supplementary Figure S5). To subtract background and quantify the effect of PlaB treatment on synthesis of each miRNA, the mean T>C conversion ratio of (*PlaB* − *Control*):(*DMSO* − *Control*) was calculated for each miRNA.

For classification by genomic location, miRNAs were assigned to genomic location using miRIAD (https://www.miriad-database.org) (49), with manual annotation using UCSC Genome Browser where necessary. miRNA genomic location was classified as intronic/exonic, lncRNA/protein coding or other. miRNAs for which an identical mature miRNA sequence mapped to >1 genomic location were included if these locations had the same context, but excluded from further analysis if they were different. UCSC Genome Browser was also used to determine the distance of miRNAs from the transcription start site (TSS). MirGeneDB 2.1 was used to establish the presence of *cis*-acting elements promoting Microprocessor cleavage.

### Statistical analysis

All quantitative data represent mean of at least three independent experiments, with error bars showing standard deviation. Western and northern blot images are representative of at least three independent experiments. Statistical significance was determined using GraphPad Prism software by Student’s *t*-test (single comparisons) or one-way ANOVA (multiple comparisons). * indicates *P*< 0.05. Two-way ANOVA and linear mixed-model fit of SLAMseq data was carried out in R.

## Results

### Inhibition of splicing reduces the level of unspliced endogenous, but not ectopically expressed, pri-miR-122

**Figure 1.**
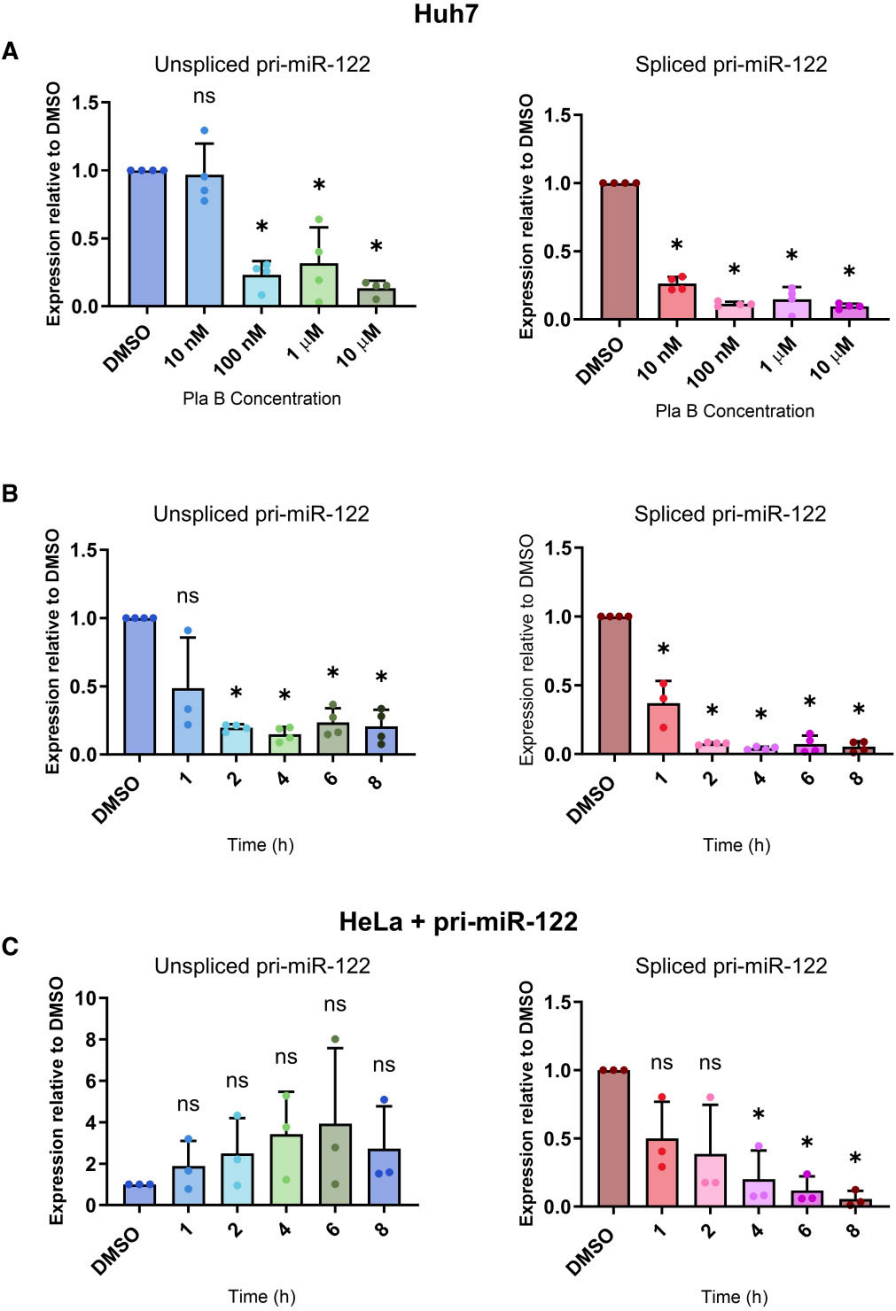
The SF3B1 inhibitor PlaB reduces the level of unspliced endogenous pri-miR-122. (**A**) Huh7 cells were treated with varying concentrations of PlaB, or equal volumes of DMSO as control, for 4 h. Total RNA was extracted and analysed by RT-qPCR with primers specific to unspliced or spliced pri-miR-122. Data were normalized to actin mRNA and shown relative to a DMSO control. (**B**) As panel (A), except that Huh7 cells were treated with 1 μM PlaB for varying time intervals. (**C**) As panel (B), except that HeLa cells transfected with a pri-miR-122 plasmid were treated with 1 μM PlaB for varying time intervals. Data represent mean of at least three independent experiments, with error bars showing standard deviation (SD). *P< 0.05; n.s., not significant.

To investigate the effects of splicing on pri-miR-122 processing and mature miR-122 production, we treated the human hepatocyte cell line Huh7, in which miR-122 is highly expressed (34), with PlaB. PlaB is a natural product-derived compound that targets the SF3B1 component of the U2 snRNP (36). SF3B1 stabilizes interactions between U2 snRNA and the BP in the pre-spliceosomal A complex, and PlaB functions by blocking conformational changes required for this recognition (50). Use of a chemical inhibitor allowed us to analyse the effects of splicing inhibition at early time points and thus minimized the likelihood of secondary effects. Huh7 cells were treated with different concentrations of PlaB, and total RNA was isolated after 4 h treatment. RT-qPCR analysis showed the expected decrease in spliced pri-miR-122 compared to DMSO control treatment, but unexpectedly we also observed a strong, concentration-dependent, decrease in unspliced pri-miR-122 (Figure 1A). The PlaB-mediated decrease in unspliced and spliced pri-miR-122 was also time-dependent (Figure 1B). Subsequent experiments were carried out using 1 μM PlaB for 4 h, as both unspliced and spliced pri-miR-122 were maximally decreased under these conditions (Figure 1B), and this allowed comparison with previous work on the effects of SF3B1 inhibition using the same conditions (17,51,52). The PlaB-mediated decrease in both unspliced and spliced pri-miR-122 was confirmed by northern blotting (Supplementary Figure S1A). Cell viability was unaffected by 1 μM PlaB up to 24 h (Supplementary Figure S1B).

To investigate whether the effects of PlaB were exclusive to the endogenous gene, we used a plasmid encoding the full pri-miR-122 transcript under the control of a heterologous promoter (HIV LTR, activated by cotransfection with a plasmid encoding the Tat transcriptional activator) (33). This plasmid was transfected into HeLa cells, which do not express endogenous pri-miR-122 (33). In this context, PlaB treatment also led to a strong decrease in spliced pri-miR-122 at later time points, but caused unspliced pri-miR-122 levels to increase, although due to experimental variation this was not statistically significant (Figure 1C). Thus, the decrease in unspliced pri-miR-122 following splice inhibition is specific to the endogenous gene. The effects of an alternative chemical inhibitor of SF3B1, the bacterial metabolite derivative spliceostatin A (SSA) (53), were also assessed. Similarly to PlaB, SSA led to a decrease in unspliced pri-miR-122 in Huh7 cells (Supplementary Figure S1C), but not in HeLa cells transfected with the pri-miR-122 plasmid (Supplementary Figure S1D).

### Splicing promotes efficient transcription of pri-miR-122

**Figure 2.**
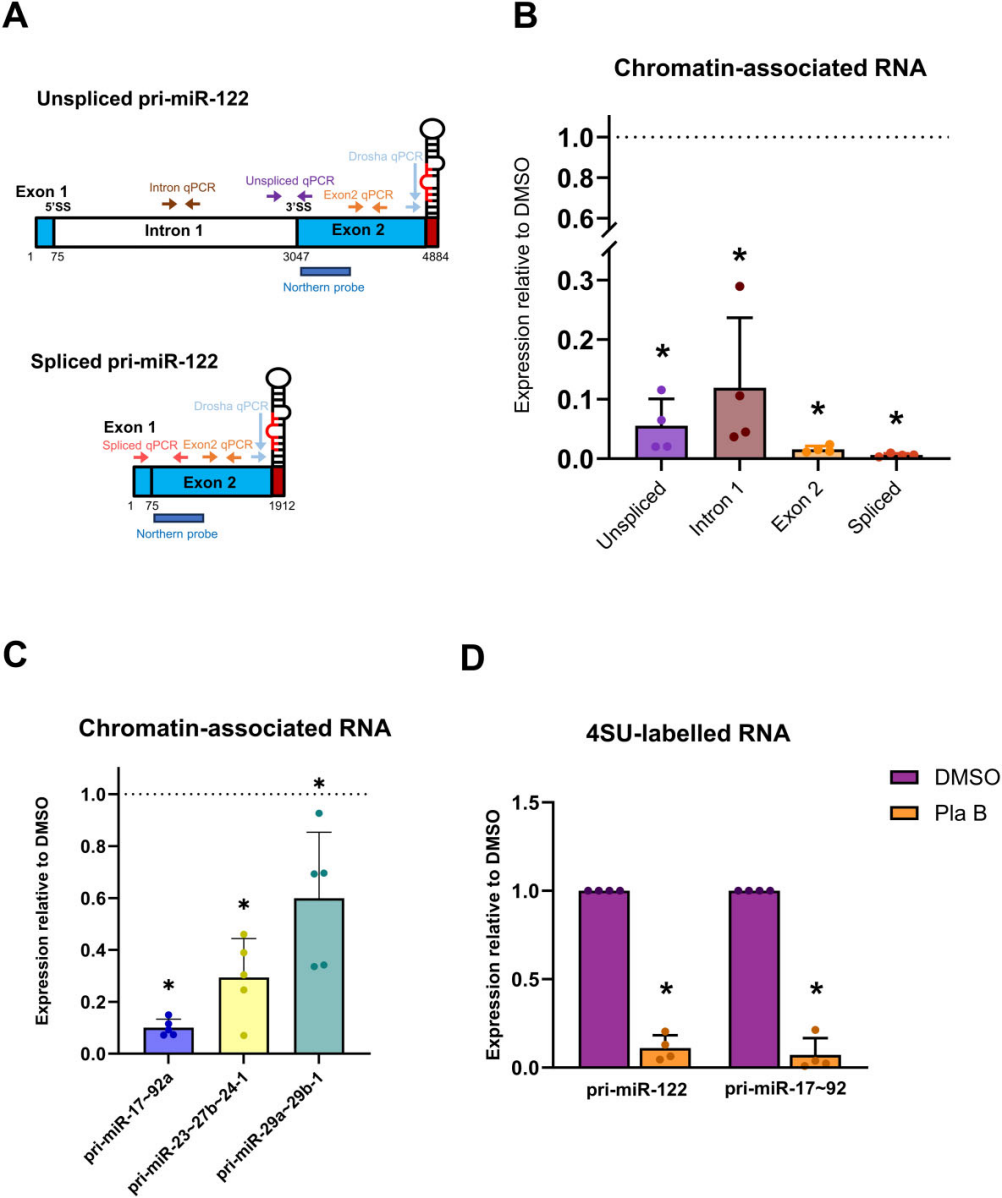
PlaB inhibits transcription of pri-miR-122 and pri-miR-17∼92a. (**A**) Diagram showing location of RT-qPCR primer pairs and northern probes on unspliced and spliced pri-miR-122. (**B**) Chromatin-associated RNA was isolated from Huh7 cells ± PlaB treatment. Pri-miR-122 was detected by RT-qPCR using the primer pairs shown and normalized to 18S rRNA. Data from PlaB-treated cells are shown relative to a DMSO-treated control. (**C**) Chromatin-associated RNA from panel (B) was analysed by RT-qPCR with primers specific to pri-miR-17∼92a, pri-miR-23b∼27b∼24-1 or pri-miR-29a∼29b-1. (**D**) Huh7 cells ± PlaB were treated with 4SU for 1 h. Total RNA was isolated, and biotinylation of 4SU followed by streptavidin pulldown was used to capture newly synthesized RNA. Pri-miR-122 and pri-miR-17∼92a levels in 4SU-labelled RNA were determined by RT-qPCR relative to 18S rRNA control and are shown relative to a DMSO-treated control. Data represent mean of at least three independent experiments, with error bars showing SD. *P< 0.05.

To establish whether the decrease in unspliced pri-miR-122 occurs at the level of transcription, we used two different methods to directly investigate effects of PlaB on nascent RNA. First, chromatin-associated RNA was biochemically isolated from Huh7 cells with and without PlaB treatment. Effective separation of chromatin was confirmed by western blotting (Supplementary Figure S2A). RT-qPCR was carried out using primers specific to intron 1, the intron 1–exon 2 junction (unspliced), the splice junction (spliced) and exon 2 (detecting both spliced and unspliced transcripts) of pri-miR-122 (Figure 2A). All sets of RT-qPCR primers showed a strong decrease in pri-miR-122 associated with chromatin following PlaB treatment compared to a DMSO control (Figure 2B), suggesting that splicing inhibition reduces pri-miR-122 transcription. To confirm that the effect of PlaB on pri-miR-122 transcription was mediated by SF3B1 inhibition, we carried out siRNA-mediated knockdown of SF3B1 (Supplementary Figure S2B). Northern blot analysis showed that SF3B1 knockdown reduced unspliced and spliced pri-miR-122 in total RNA (Supplementary Figure S2C), and RT-qPCR showed that it led to a decrease in both unspliced and spliced pri-miR-122 associated with chromatin (Supplementary Figure S2D). We therefore conclude that SF3B1 positively regulates pri-miR-122 transcription.

Additionally, we investigated whether PlaB affects transcription of two other lnc-pri-miRNAs that we have previously found to use Microprocessor cleavage to terminate transcription (33). Pri-miR-17∼92a levels were strongly decreased in chromatin-associated RNA following PlaB treatment, similarly to pri-miR-122 (Figure 2C). We also observed a decrease in chromatin-associated pri-miR-29a∼29b-1 following PlaB treatment in Huh7 cells, but this was comparatively much smaller (Figure 2C). PlaB also moderately reduced the level of chromatin-associated pri-miR-23b∼27b∼24-1, a protein-coding pri-miRNA hosting three miRNAs in an intron (Figure 2C), indicating that effects on transcription are not unique to lncRNAs.

As an alternative approach to analysis of newly transcribed RNA, we employed metabolic RNA labelling. Huh7 cells ± PlaB were treated with 4SU for 1 h, allowing 4SU incorporation into newly synthesized RNA. Subsequent isolation, biotinylation of 4SU and capture on streptavidin beads allowed nascent RNA analysis by RT-qPCR. Consistent with analysis of chromatin-associated RNA, PlaB strongly decreased the level of nascent unspliced pri-miR-122 (Figure 2D). RT-qPCR analysis of nascent pri-miR-17∼92a in the same 4SU-labelled RNA samples also showed a strong decrease following PlaB treatment relative to a DMSO control (Figure 2D). This supports our conclusion that SF3B1 is required for efficient transcription of the lncRNAs pri-miR-122 and pri-miR-17∼92a.

### Splice inhibition reduces pre-miR-122 but not mature miR-122

**Figure 3.**
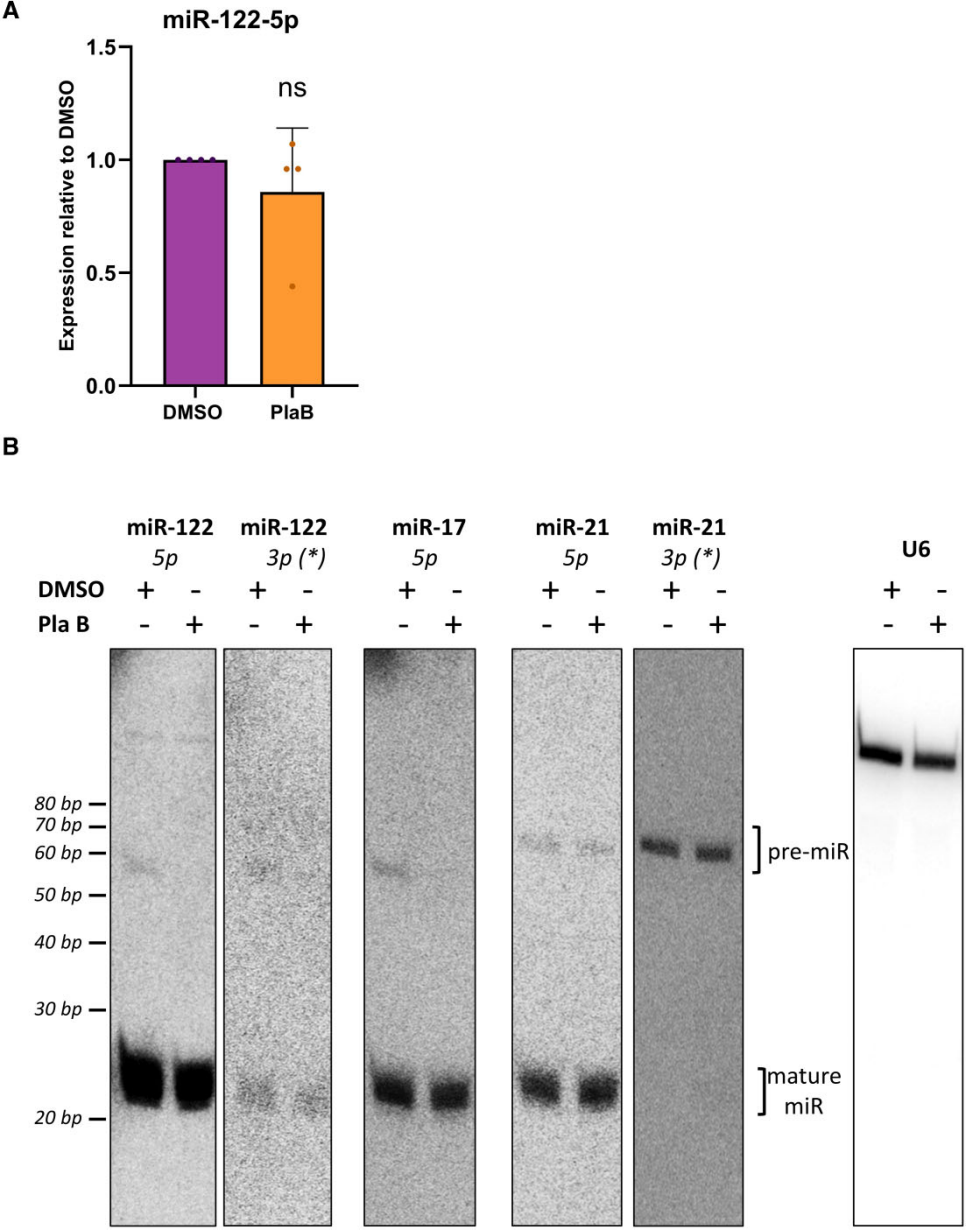
PlaB reduces the level of pre-, but not mature, miR-122 and miR-17. (**A**) Total RNA was extracted from Huh7 cells ± PlaB. Mature miR-122 was analysed by RT-qPCR, normalized to U6 snRNA and shown relative to a DMSO-treated control. Data represent mean of three independent experiments, with error bars showing SD. n.s. indicates not significant. (**B**) Total RNA extracted from Huh7 cells ± PlaB was size fractionated and the resulting small RNA was analysed by northern blot. ^32^P end-labelled oligonucleotide probes specific to the indicated mature miRNA sequences were used to detect pre- and mature miRNAs over successive probing of the same membrane. U6 snRNA was used as a loading control. Images shown are representative of at least three independent experiments.

To establish whether the strong decrease in pri-miR-122 transcription affected the expression of mature miR-122, Huh7 cells were treated with PlaB and mature miR-122 levels measured by RT-qPCR. Total miR-122 was unchanged following PlaB treatment (Figure 3A). Endogenous mature miR-122 levels were also unaffected by treatment with the SF3B1 inhibitor SSA (Supplementary Figure S3A). In contrast, mature miR-122 expressed from the pri-miR-122 plasmid in HeLa cells tended to increase when splicing was inhibited by SSA, although this was not statistically significant due to experimental variation. This may suggest competition between splicing and exonic miRNA biogenesis in this plasmid-encoded context (Supplementary Figure S3B).

Mature miRNAs are notably stable, with miR-122 having an estimated half-life between 4 and 10 days for different isoforms found in mouse liver (54). Therefore, we considered that the lack of detectable change in mature miR-122 levels after a relatively short 4 h PlaB treatment, or 8 h SSA treatment, despite decreased pri-miR-122 transcription, might be due to this high stability. Pre-miRNAs are the initial product of pri-miRNA cleavage by the Microprocessor; they are rapidly processed by Dicer into mature miRNAs (5,38). Thus, they should provide a more sensitive readout of synthesis perturbation than their mature counterparts. We therefore used northern blotting to investigate pre-miR-122 expression. Pre-miRNAs produce two mature miRNAs designated 5p and 3p according to the side of the hairpin they are produced from, of which one is the stable mature miRNA (5p for miR-122) and one is a rapidly degraded molecule, originally designated miR* (5,38), although the 5p/3p nomenclature is now preferred. Using a probe complementary to miR-122-5p, we observed no change in the mature miRNA following PlaB treatment (Figure 3B), confirming the results of RT-qPCR analysis (Figure 3A). miR-122-5p migrated as 20–22-nt bands representing 3′ isoforms (54,55), all of which were similarly unaffected by PlaB treatment. In contrast, pre-miR-122, also detected by the miR-122-5p probe (∼60 nt), was strongly decreased in Huh7 cells treated with PlaB (Figure 3B). Pre-miR-122 was also detected, and showed the same decrease following PlaB treatment, when the membrane was re-probed with an oligonucleotide specific to the 3′ arm of the hairpin (miR-122-3p, Figure 3B). Together, these results indicate that the PlaB-driven decrease in pri-miR-122 transcription leads to reduced production of pre-miR-122. It is likely that this would also result in reduced synthesis of mature miR-122, but no change is detectable at the level of the highly stable mature miRNA molecule.

To investigate the effects of PlaB on biogenesis of a second miRNA that shows PlaB-mediated transcription inhibition, the membrane was stripped and re-probed with a radiolabelled probe specific to miR-17-5p. Similarly to miR-122, mature miR-17-5p was unaffected by PlaB but pre-miR-17 was strongly decreased, suggesting that transcription inhibition by PlaB has similar effects on miR-17 biogenesis (miR-17-5p, Figure 3B). We also used probes to detect miR-21-5p on the same membrane, chosen as an miRNA that is highly expressed in liver (54,56). miR-21-5p is mainly processed from an unspliced transcript, although it can also be generated from readthrough transcripts of the upstream VMP1 gene (57) and was not identified as Microprocessor-terminated in our previous analysis (33). Both pre- and mature miR-21 were unaffected by PlaB (miR-21-5p and miR-21-3p probes, Figure 3B), indicating that the inhibitory effects of splice inhibition on miRNA biogenesis are not universal. Finally, the membrane was re-probed for U6 snRNA as a loading control, which showed no effect of PlaB (Figure 3B).

### Global analysis of the effects of splicing inhibition on miRNA synthesis

**Figure 4.**
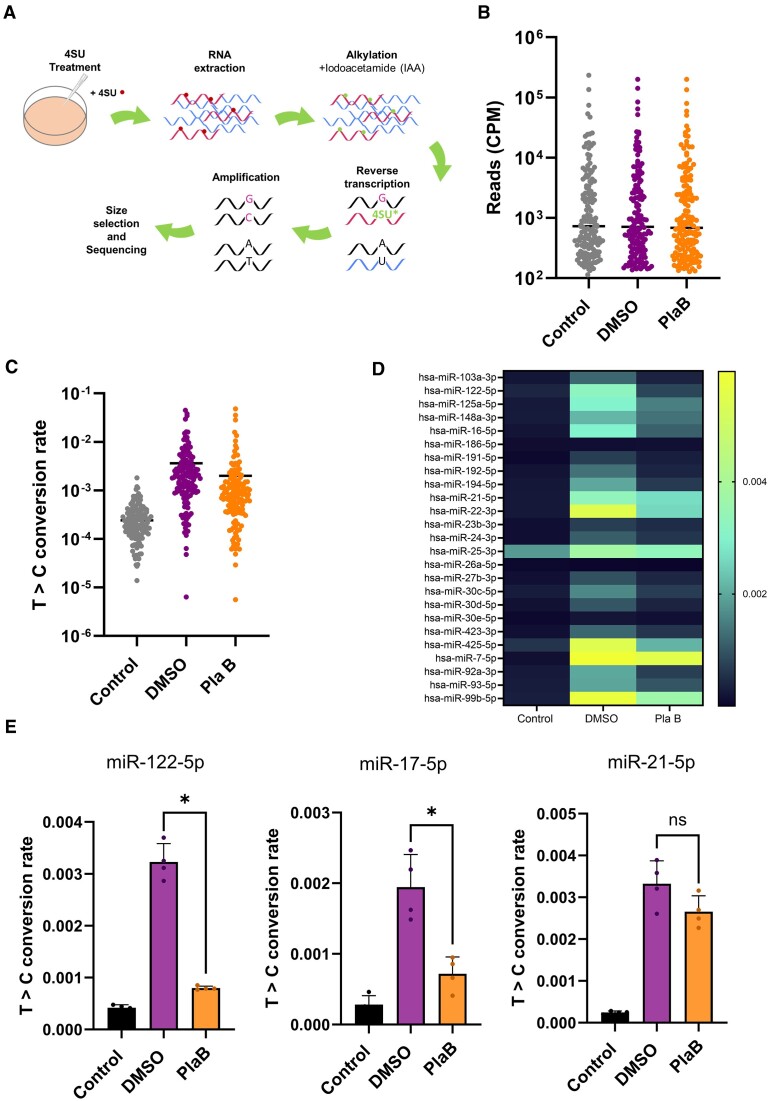
SLAMseq shows a global decrease in miRNA biogenesis following PlaB treatment. (**A**) Schematic diagram showing the SLAMseq methodology. 4SU incorporated into newly synthesized miRNA is shown as a dot. (**B**) Read counts of miRNAs in SLAMseq datasets from *Control* (DMSO, no 4SU), *DMSO* (DMSO + 4SU) and *PlaB* (PlaB + 4SU) conditions, shown as CPM for all miRNAs above the 100 CPM threshold (Initial miRNA Dataset). Two-way ANOVA showed no difference in read counts between treatments (*P*= 0.9966) but, as expected, showed difference between miRNAs (*P*< 0.0001). (**C**) T>C conversion rates for all miRNAs with >100 CPM in *Control*, *DMSO* and *PlaB* conditions (Initial miRNA Dataset). Two-way ANOVA showed that both treatment (*P*< 0.0001) and miRNA (*P*< 0.0001) as well as the treatment × miRNA interaction (*P*< 0.0001) had a significant effect on T>C conversion rates. miRNAs for which T>C conversion rate = 0 were excluded from the plot due to the log scale (21 miRNAs in *Control*, 4 miRNAs in *DMSO*, 7 miRNAs in *PlaB*). (**D**) As panel (C), except that heatmap shows T>C conversion rates for individual miRNAs, and only data for the 25 most highly expressed miRNAs are shown. (**E**) T>C conversion rates are shown for miR-122-5p, miR-17-5p and miR-21-5p in the three conditions. All data represent mean of four independent experiments, with error bars showing SD. *P< 0.05; n.s., not significant.

Our results demonstrate that pri-miR-122 and pri-miR-17∼92a show reduced transcription, leading to reduced production of pre-miRNA, following PlaB treatment, whereas pre-miR-21 synthesis is unaffected (Figures 2 and 3). We hypothesized that synthesis of mature miR-122 and miR-17 is also inhibited, but cannot be detected at the level of total RNA (Figure 3). To characterize the effects of splicing on biogenesis of mature miRNAs on a global scale, we used the SLAMseq approach that was recently adapted by the Ameres group for analysis of newly synthesized mature miRNAs (38). SLAMseq uses 4SU labelling of newly synthesized RNA, followed by a chemical conversion of 4SU to a form that is base-paired to G instead of A during reverse transcription. This means that at all positions where a 4SU nucleotide is incorporated in newly synthesized RNA, G is incorporated during reverse transcription, and modified bases are subsequently detected as T>C conversions in a sequencing reaction (Figure 4A) (44). Informed by preliminary experiments, we carried out all SLAMseq experiments at a single 4 h time point with a 500 μM concentration of 4SU, which showed no effects on cell viability at this time (Supplementary Figure S4A), to maximize the detection of labelled miRNAs while minimizing potential secondary effects of longer term inhibition of splicing. Total RNA was extracted from Huh7 cells with three different treatments: (a) DMSO treatment for 4 h with no 4SU as a control for background T>C conversion (*Control*); (b) DMSO treatment for 4 h with 4SU included throughout (*DMSO*); and (c) PlaB treatment for 4 h with 4SU included throughout (*PlaB*). Following chemical conversion of total RNA, small RNA-seq was carried out (Figure 4A). We obtained four independent datasets, which were highly reproducible at the level of read counts and T>C conversions (Supplementary Figure S4B and C, and Supplementary Table S4).

T>C conversions within mature miRNA features were quantified via SLAM-DUNK (45), using the modified SLAMseq pipeline described earlier. Due to concerns that many reported human miRNAs are false positives (58), analysis was limited to an ‘Initial miRNA Dataset’ of miRNAs expressed at >100 CPM and present in the manually curated MirGeneDB 2.1 (48) (Supplementary Figure S5). The average read counts for individual miRNAs in this dataset were similar between all three conditions (Figure 4B and Supplementary Figure S6), indicating that neither 4 h 4SU nor PlaB treatment affected the level of miRNAs. In contrast, T>C conversion rates were higher in the 4SU-containing samples (*DMSO* and *PlaB*) compared to the no 4SU control (*Control*), indicating that the SLAMseq protocol was successful in labelling newly synthesized mature miRNAs (Figure 4C). Principal component analysis of T>C conversion rate indicated that biological replicates for each condition clustered (Supplementary Figure S7), including two libraries that required re-amplification (*DMSO A* and *DMSO B*).

Comparison of average T>C conversion rates in PlaB treatment to the DMSO control in the 4SU-treated samples showed a trend to decreased synthesis of mature miRNAs following PlaB treatment (Figure 4C). Heatmap analysis of T>C conversion rates in the three conditions for the 25 most highly expressed miRNAs showed overall low background in the no 4SU condition (*Control*), with variable T>C conversion rates in cells treated with DMSO + 4SU (*DMSO*), suggesting variations in rate of synthesis or in labelling efficiency between different miRNAs in Huh7 cells (Figure 4D). Most miRNAs showed reduced T>C conversion rate following PlaB + 4SU treatment (*PlaB*) compared to *DMSO*, although the extent of reduction varied between the miRNAs (Figure 4D). The same trends were apparent when all miRNAs in the Initial miRNA Dataset were considered (Supplementary Figure S8). These results suggest that PlaB-mediated inhibition of splicing results in a global, but not universal, reduction in miRNA biogenesis.

Detailed analysis of T>C conversion rates for individual miRNAs showed decreased synthesis of mature miR-122-5p and miR-17-5p, but not miR-21-5p, following PlaB treatment (Figure 4E). This was similar to the effect of PlaB on their pre-miRNAs observed by northern blot (Figure 3B), indicating that changes in pre-miRNA level correspond to changes in mature miRNA synthesis following PlaB treatment. This confirms that SLAMseq allows detection of rapid changes in mature miRNA synthesis that are not detectable at the level of total miRNA (Figure 3), presumably due to the high stability of the mature miRNA. PlaB treatment also led to a moderate decrease in synthesis of miR-23b-3p, whereas miR-29a-3p synthesis was not significantly affected (Supplementary Figure S9), in line with the previously observed effects of PlaB of transcription of their parental pri-miR-23b∼27b∼24-1 and pri-miR-29a∼29b-1 genes (Figure 2C). This suggests that the effects of PlaB on miRNA biogenesis are largely mediated at the level of transcription.

### Effects of splicing inhibition on miRNA biogenesis are not influenced by genomic location or *cis*-elements that promote Microprocessor cleavage

Next, we wished to determine whether genomic context influenced the effects of splicing inhibition on miRNA biogenesis. miRNAs in our ‘Initial miRNA Dataset’ were classified by genomic location as intronic/exonic and protein-coding/lncRNA (Supplementary Figure S10A–D), with miRNAs with mixed location excluded from further analysis. The distribution of miRNAs between these categories was similar to that observed in other studies analysing genomic location of all human miRNAs (19–21). To ensure that quantification of the effects of splicing on miRNA biogenesis was limited to miRNAs with confident annotation that were labelled above background, we introduced additional filtering steps, reducing the number of miRNAs analysed to 102 in our Final miRNA Dataset (Supplementary Table S4 and Supplementary Figure S5). To compare the effect of PlaB on synthesis of different categories of miRNA, *Control* (no 4SU) T>C conversion rate for each miRNA was subtracted from *DMSO* or *PlaB* (+4SU) T>C conversion rate to remove background. Comparison of background subtracted PlaB T>C conversions to background subtracted DMSO T>C conversions indicated that PlaB treatment reduces synthesis of most miRNAs (Supplementary Figure S11).

**Figure 5.**
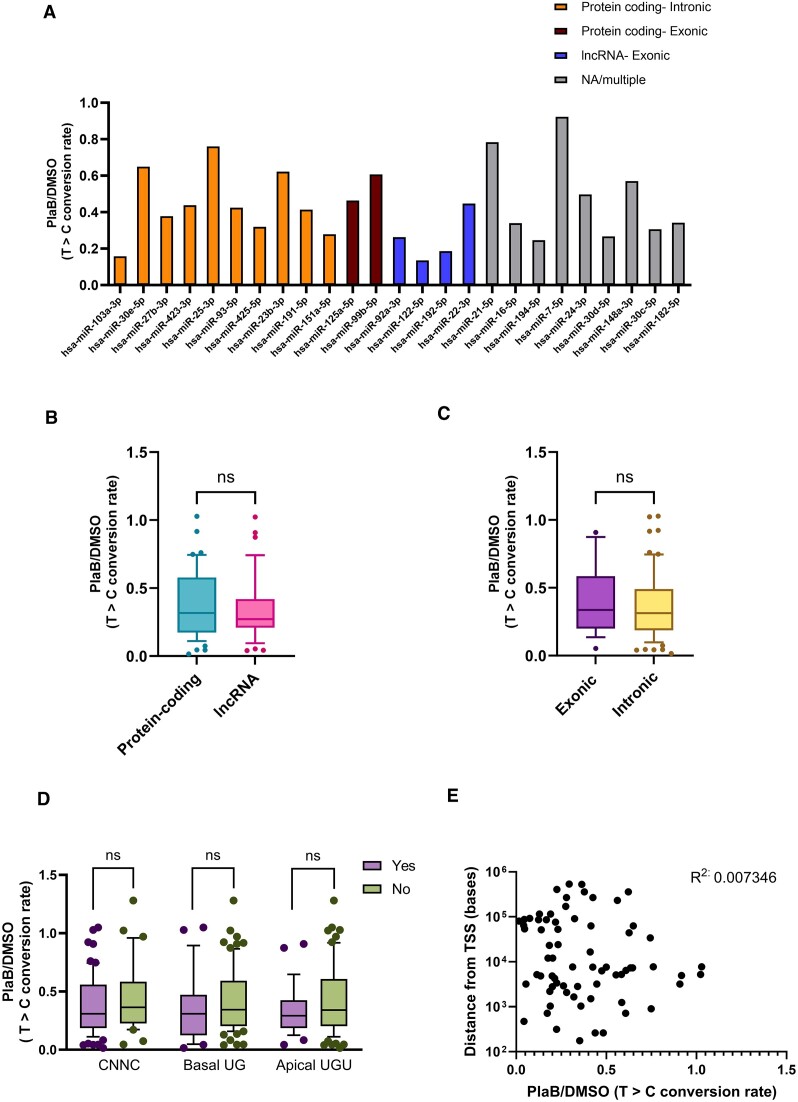
Genomic location does not influence the effect of PlaB on miRNA biogenesis. (**A**) Effects of PlaB on biogenesis of the 25 most highly expressed miRNAs. miRNAs are grouped according to genomic location. For each genomic location, miRNAs are ordered from highest to lowest expression. (**B**) Comparison of the effect of PlaB on biogenesis of all miRNAs in the filtered dataset from exonic or intronic locations. (**C**) As panel (B), except that miRNAs were grouped by lncRNA or protein-coding gene location. (**D**) As panel (B), except that miRNAs were grouped by the presence or absence of the CNNC, basal UG or apical UGU *cis*-acting motifs. (**E**) Scatterplot showing effect of PlaB on miRNA biogenesis relative to distance from TSS. For all data, the effect of PlaB was determined by T>C conversion rate in (*PlaB* − *Control*)/(*DMSO* − *Control*) based on mean values of four independent experiments for each miRNA. Box plots in panels (B)–(D) show median as a line, with 10th and 90th centiles forming the box, and outliers shown as individual data points. n.s., not significant.

To quantify the change in miRNA biogenesis following PlaB treatment for individual miRNAs, background subtracted PlaB T>C conversion rate was then divided by that of DMSO. Comparison of the effects of PlaB on synthesis of the 25 most highly expressed miRNAs showed a general decrease, but with individual miRNAs affected to a varying extent (Figure 5A). miRNAs expressed from different genomic locations also showed a range of response to PlaB, with no obvious association between the genomic location and the extent of repression (Figure 5A). Interestingly, comparison of the effect of PlaB on synthesis of all miRNAs in the Final miRNA Dataset grouped by exonic or intronic location showed no significant difference between the two classes (Figure 5B). There was also no significant difference in the effect of PlaB on miRNAs hosted in lncRNA genes compared to those hosted in protein-coding genes (Figure 5C). miRNAs were also grouped according to the presence or absence of known *cis*-acting features that modulate Microprocessor cleavage (7), none of which affected the response to splice inhibition (Figure 5D). Finally, we tested whether the distance between a mature miRNA and its TSS affects the response to PlaB, and found no correlation (Figure 5E). We conclude that miRNAs in all genomic locations show a trend towards reduced synthesis following PlaB treatment, although the extent of this reduction is variable between individual miRNAs. The lack of correlation with exonic/intronic location or features associated with Microprocessor cleavage supports the hypothesis that the effects of PlaB on miRNA biogenesis are mediated primarily at the level of transcription.

### Inhibition of miR-122 biogenesis by PlaB is independent of the pri-miR-122 3′SS

**Figure 6.**
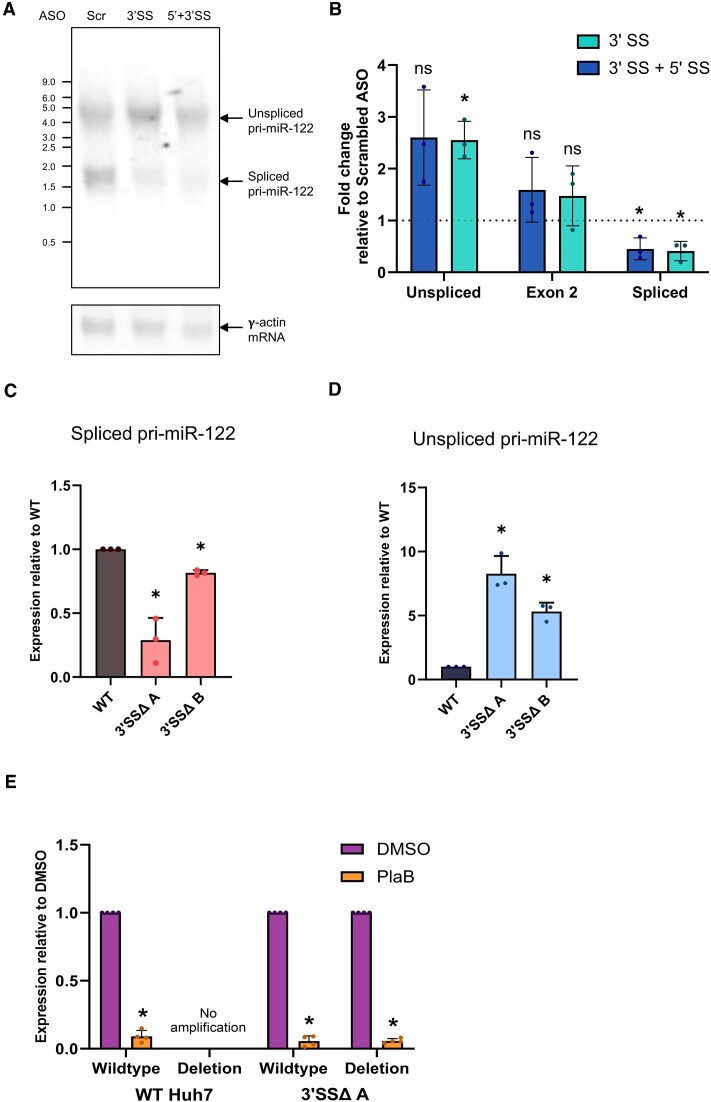
Pri-miR-122 3′SS inhibition or mutation does not inhibit transcription. (**A**) Northern blot showing reduction of spliced, but not unspliced, pri-miR-122 in total RNA extracted from Huh7 cells following transfection of ASOs targeting the 3′SS, or 5′SS + 3′SS, compared to a scrambled control ASO. γ-Actin mRNA is shown as a loading control. (**B**) Chromatin-associated RNA was extracted from ASO-transfected cells and analysed by RT-qPCR with primer pairs specific to pri-miR-122, as shown in Figure 2A, relative to GAPDH pre-mRNA. (**C**) CRISPR/Cas9 modification was used to delete the region spanning the branch point, polypyrimidine tract and 3′SS of pri-miR-122 in Huh7 cells. Total RNA was extracted from two clonal cell lines heterozygous for the mutation (3′SSΔ A and 3′SSΔ B), and WT Huh7 cells cultured in parallel, and analysed by RT-qPCR with primers specific to spliced pri-miR-122. Pri-miR-122 was normalized to 18S rRNA and is shown relative to WT Huh7. (**D**) As panel (C), except that unspliced pri-miR-122 was analysed by RT-qPCR. (**E**) WT Huh7 cells or heterozygous 3′SSΔ clone A cells were treated with PlaB and total RNA extracted. RT-qPCR with primers specific to the WT or mutant (deletion) unspliced pri-miR-122 was used to determine the effects of PlaB relative to a DMSO-treated control, with data normalized to 18S rRNA. Northern blot is representative of two independent experiments and all other data represent mean of at least three independent experiments, with error bars showing SD. *P< 0.05; n.s., not significant.

To directly investigate the role of splicing of the single pri-miR-122 intron, we designed ASOs to target the 5′SS and 3′SS of pri-miR-122 and introduced them into Huh7 cells. Northern blot analysis of total RNA from transfected cells indicated that the 3′SS ASO alone, or in combination with the 5′SS ASO, led to a reduction in spliced pri-miR-122 without a decrease in unspliced pri-miR-122 (Figure 6A), in contrast to the decrease in both unspliced and spliced pri-miR-122 seen with SF3B1 inhibition (Supplementary Figure S1A). No additional bands were observed, making it very unlikely that cryptic splicing occurs when canonical pri-miR-122 splicing is inhibited. RT-qPCR analysis of chromatin-associated RNA showed that ASO-mediated pri-miR-122 splicing inhibition led to the expected decrease in spliced pri-miR-122 (Figure 6B). In agreement with the northern blot, ASO treatment did not affect overall pri-miR-122 transcription, as shown by exon 2 primers that bind to both unspliced and spliced pri-miR-122 (Figures 2A and 6B). Unspliced pri-miR-122 associated with chromatin was slightly increased (Figure 6B), confirming that ASO treatment inhibits splicing without also inhibiting transcription, in contrast to the effects of PlaB.

Next, we used CRISPR/Cas9 to delete a region spanning the branch point, polypyrimidine tract and 3′SS in pri-miR-122 (Supplementary Figure S12A). Huh7 cells were previously shown to contain two copies of the pri-miR-122 locus (59). Although we were not successful in generating a homozygous pri-miR-122 deletion mutant, we successfully generated two clones with a heterozygous deletion mutation, designated 3′SSΔ (Supplementary Figure S12B). The two 3′SSΔ clonal populations were cultured in parallel to WT Huh7 cells, and levels of spliced and unspliced pri-miR-122 in total RNA were determined by RT-qPCR. As expected, both 3′SSΔ cell lines showed a reduction in spliced pri-miR-122 compared to WT (Figure 6C), although the level of reduction varied between the clones. However, we observed higher levels of unspliced pri-miR-122 in 3′SSΔ relative to WT cells (Figure 6D). Taken together with the ASO data, this indicates that direct inhibition of pri-miR-122 splicing by deletion or inhibition of the 3′SS does not reproduce the inhibitory effects of PlaB on pri-miR-122 transcription.

The heterozygous nature of the 3′SSΔ cells allowed us to directly investigate the response of WT and 3′SSΔ alleles to PlaB treatment in the same cells. We selected 3′SSΔ cell line A for further analysis, due to the stronger reduction in spliced pri-miR-122 observed in this clone than in clone B (Figure 6C). RT-qPCR primers were designed to specifically amplify either WT or 3′SSΔ unspliced pri-miR-122 in total RNA extracted from WT or mutant cells. Both WT and 3′SSΔ unspliced pri-miR-122 transcripts showed a similar strong reduction following PlaB treatment (Figure 6E). Overall, these results indicate that the role of SF3B1 in promotion of pri-miR-122 transcription is not dependent on a functional 3′SS.

### Effects of splicing on miR-122 biogenesis independent of transcription inhibition are relatively minor

**Figure 7.**
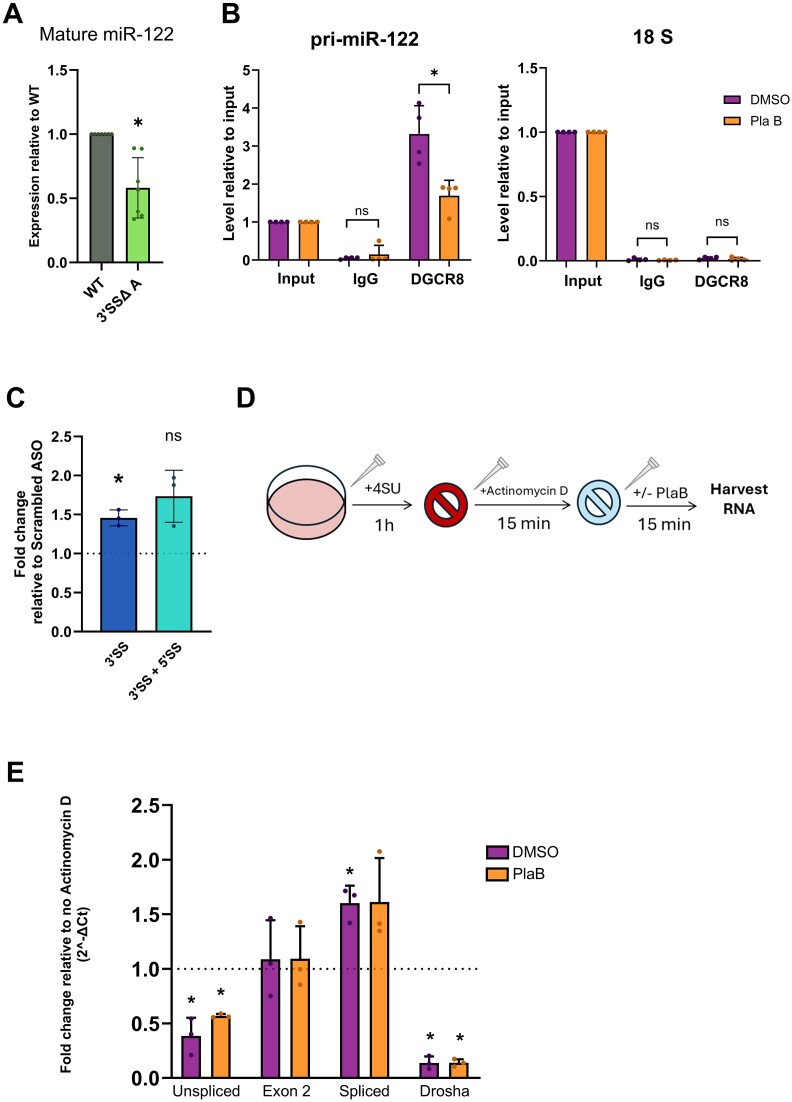
Splicing promotes cotranscriptional, but not post-transcriptional, Microprocessor cleavage of pri-miR-122 independent of transcription. (**A**) Mature miR-122 levels in 3′SSΔ A were determined by RT-qPCR, normalized to U6 snRNA and shown relative to WT Huh7 cells cultured in parallel. (**B**) RIP was carried out using an antibody to endogenous DGCR8 in Huh7 cells ± PlaB. RT-qPCR with primers 5′ of the pre-miR-122 hairpin was used to measure pri-miR-122 in DGCR8 RIP, or an IgG control, relative to 10% input. 18S rRNA was measured by RT-qPCR as a control for non-specific binding. (**C**) Chromatin-associated RNA was extracted from ASO-transfected cells and analysed by RT-qPCR with primers spanning the Drosha cleavage site on pri-miR-122, relative to GAPDH pre-mRNA. (**D**) Diagram showing the approach used to investigate pri-miR-122 processing following transcription inhibition in panel (E). (**E**) 4SU-labelled RNA was isolated from Huh7 cells ± PlaB treatment following Act D treatment, and analysed by RT-qPCR using primer pairs shown in Figure 2A. RT-qPCR with different sets of primers for pri-miR-122 was used to detect changes in 4SU-labelled RNA from DMSO- and PlaB-treated cells relative to a no Act D control, normalized to 18S rRNA. Data represent mean of at least three independent experiments, with error bars showing SD. *P< 0.05; n.s., not significant.

Finally, we wished to investigate whether disruption of pri-miR-122 splicing has additional effects on miR-122 biogenesis independent of the effects on transcription. RT-qPCR analysis of mature miR-122 levels in 3′SSΔ clone A compared to WT showed a significant but variable decrease in miR-122 when the splice site was mutated (Figure 7A), suggesting that inhibition of splicing reduces miR-122 biogenesis even when transcription is not inhibited. Next, we used RIP to detect DGCR8 interaction with pri-miR-122. Using RT-qPCR primers just upstream of the pre-miR-122 hairpin, we observed a strong enrichment of pri-miR-122, but not 18S rRNA, in the DGCR8 RIP but not an IgG control (Figure 7B), confirming that the RIP was specific. When normalized to input RNA to take account of the effects on pri-miR-122 transcription, PlaB treatment led to a 2-fold decrease in Microprocessor-associated pri-miR-122 (Figure 7B). Third, we designed RT-qPCR primers that span the site of Drosha cleavage in pri-miR-122 (Figure 2A). We used these primers to look at changes in the signal across the Drosha cleavage site in chromatin-associated RNA after splice inhibition with ASOs, which we previously showed had no effect on pri-miR-122 transcription (Figure 6B). We observed a small increase, suggesting that ASO-mediated splicing inhibition may lead to reduced cleavage by Microprocessor (Figure 7C). However, exon 2 RT-qPCR signal, representing total chromatin-associated pri-miR-122, was also slightly, although not significantly, increased by ASO transfection (Figure 6B), suggesting that the decrease in Microprocessor cleavage observed is minor.

As an alternative approach to directly investigate pri-miR-122 processing without confounding effects of transcription, we treated Huh7 cells with 4SU for 1 h to label newly synthesized RNA and then treated them with Act D for 15 min to block transcription before treating with PlaB for 15 min (Figure 7D). Following biotinylation and isolation of 4SU-labelled RNA, we found that Act D led to a decrease in unspliced pri-miR-122 (Figure 7E), confirming that unspliced pri-miR-122 is unstable (33). Spliced pri-miR-122 was increased by Act D, suggesting that splicing of the 4SU-labelled unspliced pri-miR-122 is occurring more rapidly than decay of existing spliced pri-miR-122. Exon 2 RT-qPCR signal, which represents both unspliced and spliced pri-miR-122, was unaffected by the short Act D treatment. Interestingly, RT-qPCR across the Drosha cleavage site showed a larger decrease than unspliced pri-miR-122 in Act D-treated cells, indicating that Microprocessor cleavage occurs faster than splicing when transcription is inhibited. Treatment with PlaB following the Act D transcriptional block did not significantly affect RT-qPCR signal with any pri-miR-122 primers, although there was a slight but variable increase in unspliced pri-miR-122 (Figure 7E) suggesting that inhibition of splicing is beginning to occur. However, the lack of effect of PlaB on RT-qPCR signal across the Drosha cleavage site shows that Microprocessor cleavage was unaffected by PlaB at this early time point (Figure 7E). As this experiment was performed under conditions of transcription inhibition, both splicing and Microprocessor cleavage during the PlaB treatment would occur post-transcriptionally following release of pri-miR-122 from the chromatin. In this context, we observe no effect of splicing inhibition on Microprocessor cleavage, in contrast to the small effects of splicing on cotranscriptional Microprocessor recruitment and cleavage (Figure 7A–C).

## Discussion

In this study, we used both a detailed analysis of transcription and processing of the pri-miR-122 gene and a global analysis of newly synthesized miRNAs to establish the effects of splicing inhibition on miRNA synthesis in cultured human cells. We established that splicing inhibition using the SF3B1 inhibitor PlaB strongly and rapidly reduced transcription of the pri-miR-122 lncRNA, with associated decrease in mature miR-122 synthesis. Genome-wide analysis using SLAMseq allowed us to directly investigate changes in miRNA synthesis over a few hours of PlaB treatment, avoiding potential secondary effects of long-term splicing inhibition, or lack of sensitivity due to the high stability of mature miRNAs. The SLAMseq analysis showed that production of most miRNAs was decreased by PlaB treatment. Interestingly, we found that miRNAs in intronic or exonic context were not differentially regulated by splicing inhibition.

Importantly, despite northern blotting showing that pre-miR-122 was almost entirely lost following PlaB treatment (Figure 3B), we did not observe any significant change in the level of mature miR-122 by RT-qPCR or northern blot (Figure 3A and B). This was likely due to the long half-life of miR-122 and the presence of a stable pool of the mature miRNA (54). While specific miRNAs are known to be rapidly turned over in specific circumstances, for example via target-directed miRNA degradation, or in neurons (60), most animal miRNAs in most cells are highly stable (38,61). Most studies examining changes in miRNA synthesis rely on quantification of miRNAs in total RNA (either by RNA-seq or by RT-qPCR). These studies may be limited by low sensitivity to changes in miRNA biogenesis, particularly decreases, and/or by a requirement for long-term perturbation of cells to detect changes, which increases potential for secondary effects. SLAMseq addresses these problems by specifically analysing newly synthesized miRNAs. While metabolic labelling and isolation of newly synthesized RNA has also been used to analyse miRNA metabolism by RNA-seq (62), an advantage of SLAMseq is that the chemical conversion approach allows internal normalization to the unlabelled miRNAs in the same dataset (38). This minimizes effects of sequencing bias, which is a known challenge for miRNA-seq (63), although the randomized adapter approach used in the NEXTflex library preparation and our analysis approach of comparing two conditions (*PlaB* and *DMSO*) for the same miRNA were also designed to minimize this.

We successfully applied SLAMseq to Huh7 cells, identified T>C conversions above background and were able to quantify the effects on PlaB on synthesis of 102 miRNAs that were expressed at >100 CPM and labelled above background. A very few miRNAs were excluded during filtering as T>C conversion rates were at the background level in PlaB datasets, but above background in DMSO treatment. It is possible that this filtering may have removed some miRNAs for which synthesis was very strongly inhibited or blocked by PlaB treatment from subsequent analysis. The overall distribution of miRNA reads in our libraries was similar to that expected for human liver (56), although both miR-21-5p and miR-92a-3p were more highly expressed than miR-122-5p (Supplementary Table S4), similar to previous results in the Huh7-derived cell line Huh7.5 (64,65). Total miRNA levels were unaffected by 4 h PlaB (Figure 4B and Supplementary Figure S6), demonstrating the importance of SLAMseq to detect changes in biogenesis. There are some limitations to the use of SLAMseq to analyse miRNAs, most importantly the short length of the mature miRNA that has been shown to reduce sensitivity (45). The average T>C conversion rate across miRNAs in the *DMSO* condition was only 0.35%, comparable to those obtained at similar time points by the Ameres and Shiekhatter groups at similar time points in *Drosophila* S2 cells (38) or HeLa cells (66), meaning that most miRNAs produced in the 4 h experiment would not contain a single T>C conversion. Therefore, only a proportion of the newly synthesized miRNA population is detected by measurement of T>C conversion rates, and the rate of labelling of individual miRNAs will also be influenced by U content. As discussed in the context of sequencing bias, our approach of comparing synthesis of each miRNA in PlaB and DMSO conditions allows the effects of PlaB on individual miRNAs to be determined despite differential incorporation rates and the fact that only a proportion of newly synthesized miRNAs will be sampled. However, our methodology does not allow direct comparison of biosynthesis rates of different miRNAs, for which time course analysis would be necessary.

We also attempted to use the more established approach of 4SU labelling, followed by biotinylation and streptavidin pulldown (42,43), to specifically investigate changes in production of individual miRNAs by RT-qPCR, but found that this method was prone to artefactual results when investigating mature miRNA synthesis (data not shown), likely due to the low incorporation rate of 4SU in mature miRNAs observed by SLAMseq. Despite its limitations, we therefore recommend SLAMseq as the most sensitive and reliable method of detecting miRNA biogenesis changes. As a further methodological note, we also tested various RT-qPCR methods to detect pre-miR-122 directly, including commercial kits, but did not find any that avoided contamination from pri-miR-122 (data not shown). This is not surprising given that the pre-miR-122 sequence is entirely present in pri-miR-122, and we advise caution when interpreting any pre-miRNA RT-qPCR assays. miRNA northern blot is the most reliable method of specifically analysing pre-miRNA, but it can be challenging to detect the short-lived pre-miRNA. Size fractionation of total RNA to enrich for small RNA before gel fractionation allowed us to do this. The differences in detectable level of pre-miRNA for the different miRNAs we tested (Figure 3B) suggest differences in the efficiency of Dicer processing in Huh7 cells, which warrant further exploration.

Previous analysis of the relationship between miRNA biogenesis and splicing has tended to focus on miRNAs embedded in introns or exons of protein-coding pre-mRNAs, analysed at steady state. In contrast to previous studies of the effects of splicing on miRNA biogenesis, our work has several novel aspects: (i) analysis of changes after short-term (4 h) splicing inhibition induced by a small molecule drug; (ii) direct analysis of new miRNA synthesis during the window of inhibition as opposed to changes in stable miRNA populations; and (iii) analysis of miRNAs expressed from their endogenous genomic context rather than minigene constructs. Together, these aspects helped to ensure that biologically relevant direct effects of splicing inhibition on miRNA biogenesis were detected, and are likely to explain some of the differences from earlier studies that have used longer term siRNA-mediated knockdown of splice factors and/or minigenes. However, our results largely agree with a few other studies that have shown effects of SF3B1 on levels of many miRNAs without fully establishing the mechanism (28,67–69), suggesting that these results are largely driven by a requirement for SF3B1 to promote efficient transcription of most pri-miRNAs. Interestingly, several proteins with a role in splicing have been implicated in miRNA biogenesis by promotion of transcription in *Arabidopsis thaliana* (70–72).

We observed no effect of location within the pri-miRNA (intronic, exonic, distance from TSS), gene type (lncRNA or pre-mRNA) or *cis*-elements that affect Microprocessor cleavage on the response of individual miRNAs to PlaB (Figure 5). Instead, we observed similar inhibitory effects of PlaB on pri-miRNA transcription and miRNA biogenesis for a few specific miRNAs (Figures 2 and 4E, and Supplementary Figure S9), suggesting that the effect of SF3B1 in promoting miRNA biogenesis is largely mediated at the level of transcription. Genome-wide approaches to globally characterize the effects of PlaB on pri-miRNA transcription, splicing and Microprocessor cleavage in Huh7 cells were beyond the scope of this study but will be an important area of future research. It has recently been shown that SF3B1 promotes efficient RNAPII transcription of specific genes, both by promotion of promoter-proximal pause release (52) and by prevention of premature transcription termination (17). Our results add to these genome-wide studies by showing a very strong and rapid effect of PlaB treatment on transcription of an lncRNA, despite this transcript being inefficiently spliced and its transcription terminated by Microprocessor cleavage rather than the canonical CPA method (33). The determinants of which genes are transcriptionally regulated by PlaB are currently unknown, but chromatin context has been implicated (73). Our observation that endogenous, but not plasmid-encoded, pri-miR-122 is transcriptionally inhibited by PlaB (Figure 1C) supports the possibility that promoter features and/or chromatin context may influence the response of individual genes to SF3B1 inhibition, and therefore the downstream effects on miRNA biogenesis. This will be an important area of future research.

Importantly, inhibition of pri-miR-122 3′SS recognition by both ASO transfection and CRISPR-mediated deletion did not reduce pri-miR-122 transcription, and PlaB effectively inhibited transcription of the 3′SSΔ mutant allele (Figure 6), indicating that the effect of SF3B1 on pri-miR-122 transcription is not dependent on U2 snRNP function at an active branch point and 3′SS. Analysis of pri-miR-122 processing in conditions in which splicing but not transcription is inhibited, and RIP analysis of DGCR8 recruitment, suggests that splicing can promote Microprocessor cleavage of pri-miR-122 independent of transcription regulation (Figure 7A–C), but these effects are small compared to the effect of PlaB on transcription and only occur in the context of cotranscriptional Microprocessor cleavage (Figure 7E).

Our research has implications for the development of SF3B1 inhibitors such as PlaB that show potent anti-tumour activity *in vitro* and *in vivo* (74–76). The molecular basis for selective inhibition of cancer cell growth is not fully understood, although most research has focused on characterizing differentially regulated splicing events (76). Given the important role of miRNAs in cancer (77) and the strong effects of PlaB on miRNA production that we have observed, we advise that changes in miRNAs are taken into consideration when elucidating the anti-cancer mechanism of PlaB and other SF3B1 inhibitors. Our results also provide new insights into the complexity of miRNA biogenesis, better understanding of which may offer potential avenues for therapeutic manipulation of endogenous miRNA expression. miR-122 is of particular interest as it is exceptionally highly and specifically expressed in hepatocytes (32,54,56,78), and our data contribute to understanding of factors that underlie this high expression. This may be important for therapeutic manipulation of miR-122, which has shown considerable promise in the context of HCV infection (79,80) but could also be relevant to hypercholesterolemia and hepatocellular carcinoma (35,81,82). miRNA-based backbones also show promise for therapeutic delivery of short hairpin (sh)RNAs designed to knock down endogenous mRNAs through the RNA interference pathway (83). Better understanding of the control of miRNA biogenesis has the potential to inform such approaches, particularly a recent approach based on modifying the endogenous pri-miR-122 gene to express a liver-specific shRNA (59).

In conclusion, we have found that inhibition of the splicing factor SF3B1 leads to an overall reduction in miRNA biogenesis, and have demonstrated the utility of SLAMseq in analysis of rapid changes in miRNA production that are not detectable at the level of total miRNA. For miR-122-5p, we find that this is largely a consequence of reduced transcription, although we also observe small transcription-independent effects of splicing on cotranscriptional microprocessing of the lncRNA pri-miR-122. While lncRNAs tend to contain fewer introns and to be less efficiently spliced than pre-mRNAs (16,30,31), this study demonstrates that the splicing machinery can play an important role in facilitating efficient transcription of, and miRNA production from, both protein-coding and lnc-pri-miRNAs.

## Supplementary Material

gkae505_Supplemental_Files

## Data Availability

Raw and processed RNA-seq data files from the SLAMseq experiment have been deposited at the Gene Expression Omnibus, accession number GSE240904. All other data underlying this article are available in the article or its online supplementary material.

## Notes

Present address: Aimee L. Parsons, Merck Life Sciences Limited, Gillingham, UK.

Present address: Matthew C. Heatley, Oxford Nanopore Technologies, Oxford, UK.

Present address: Athena R.G. Martin, Sygnature Discovery, Nottingham, UK.

## References

[1] Bartel D.P. Metazoan microRNAs. Cell. 2018; 173:20–51. 29570994 10.1016/j.cell.2018.03.006 PMC6091663

[2] Gebert L.F.R. , MacRae I.J. Regulation of microRNA function in animals. Nat. Rev. Mol. Cell Biol. 2019; 20:21–37. 30108335 10.1038/s41580-018-0045-7 PMC6546304

[3] Rupaimoole R. , Slack F.J. MicroRNA therapeutics: towards a new era for the management of cancer and other diseases. Nat. Rev. Drug Discov. 2017; 16:203–222. 28209991 10.1038/nrd.2016.246

[4] Diener C. , Keller A., Meese E. Emerging concepts of miRNA therapeutics: from cells to clinic. Trends Genet. 2022; 38:613–626. 35303998 10.1016/j.tig.2022.02.006

[5] Ha M. , Kim V.N. Regulation of microRNA biogenesis. Nat. Rev. Mol. Cell Biol. 2014; 15:509–524. 25027649 10.1038/nrm3838

[6] Treiber T. , Treiber N., Meister G. Regulation of microRNA biogenesis and its crosstalk with other cellular pathways. Nat. Rev. Mol. Cell Biol. 2019; 20:5–20. 30728477 10.1038/s41580-019-0106-6

[7] Auyeung V.C. , Ulitsky I., McGeary S.E., Bartel D.P. Beyond secondary structure: primary-sequence determinants license pri-miRNA hairpins for processing. Cell. 2013; 152:844–858. 23415231 10.1016/j.cell.2013.01.031 PMC3707628

[8] Treiber T. , Treiber N., Plessmann U., Harlander S., Daiß J.L., Eichner N., Lehmann G., Schall K., Urlaub H., Meister G. A compendium of RNA-binding proteins that regulate microRNA biogenesis. Mol. Cell. 2017; 66:270–284. 28431233 10.1016/j.molcel.2017.03.014

[9] Rice G.M. , Shivashankar V., Ma E.J., Baryza J.L., Nutiu R. Functional atlas of primary miRNA maturation by the Microprocessor. Mol. Cell. 2020; 80:892–902. 33188727 10.1016/j.molcel.2020.10.028

[10] Kang W. , Fromm B., Houben A.J., Høye E., Bezdan D., Arnan C., Thrane K., Asp M., Johnson R., Biryukova I. et al. MapToCleave: high-throughput profiling of microRNA biogenesis in living cells. Cell Rep. 2021; 37:110015. 34788611 10.1016/j.celrep.2021.110015

[11] Kim K. , Baek S.C., Lee Y.-Y., Bastiaanssen C., Kim J., Kim H., Kim V.N. A quantitative map of human primary microRNA processing sites. Mol. Cell. 2021; 81:3422–3439. 34320405 10.1016/j.molcel.2021.07.002

[12] Morlando M. , Ballarino M., Gromak N., Pagano F., Bozzoni I., Proudfoot N.J. Primary microRNA transcripts are processed co-transcriptionally. Nat. Struct. Mol. Biol. 2008; 15:902–909. 19172742 10.1038/nsmb.1475 PMC6952270

[13] Ballarino M. , Pagano F., Girardi E., Morlando M., Cacchiarelli D., Marchioni M., Proudfoot N.J., Bozzoni I. Coupled RNA processing and transcription of intergenic primary microRNAs. Mol. Cell. Biol. 2009; 29:5632–5638. 19667074 10.1128/MCB.00664-09 PMC2756881

[14] Fang W. , Bartel D.P. MicroRNA clustering assists processing of suboptimal microRNA hairpins through the action of the ERH protein. Mol. Cell. 2020; 78:289–302. 32302541 10.1016/j.molcel.2020.01.026 PMC7243034

[15] Herzel L. , Ottoz D.S.M., Alpert T., Neugebauer K.M. Splicing and transcription touch base: co-transcriptional spliceosome assembly and function. Nat. Rev. Mol. Cell Biol. 2017; 18:637–650. 28792005 10.1038/nrm.2017.63 PMC5928008

[16] Tilgner H. , Knowles D.G., Johnson R., Davis C.A., Chakrabortty S., Djebali S., Curado J., Snyder M., Gingeras T.R., Guigó R. Deep sequencing of subcellular RNA fractions shows splicing to be predominantly co-transcriptional in the human genome but inefficient for lncRNAs. Genome Res. 2012; 22:1616–1625. 22955974 10.1101/gr.134445.111 PMC3431479

[17] Sousa-Luís R. , Dujardin G., Zukher I., Kimura H., Weldon C., Carmo-Fonseca M., Proudfoot N.J., Nojima T. POINT technology illuminates the processing of polymerase-associated intact nascent transcripts. Mol. Cell. 2021; 81:1935–1950. 33735606 10.1016/j.molcel.2021.02.034 PMC8122139

[18] Kataoka N. , Fujita M., Ohno M. Functional association of the Microprocessor complex with the spliceosome. Mol. Cell. Biol. 2009; 29:3243–3254. 19349299 10.1128/MCB.00360-09 PMC2698730

[19] Rodriguez A. , Griffiths-Jones S., Ashurst J.L., Bradley A. Identification of mammalian microRNA host genes and transcription units. Genome Res. 2004; 14:1902–1910. 15364901 10.1101/gr.2722704 PMC524413

[20] Chang T.C. , Pertea M., Lee S., Salzberg S.L., Mendell J.T. Genome-wide annotation of microRNA primary transcript structures reveals novel regulatory mechanisms. Genome Res. 2015; 25:1401–1409. 26290535 10.1101/gr.193607.115 PMC4561498

[21] Sun Q. , Hao Q., Lin Y.-C., Song Y.J., Bangru S., Arif W., Tripathi V., Zhang Y., Cho J.-H., Freier S.M. et al. Antagonism between splicing and Microprocessor complex dictates the serum-induced processing of lnc-MIRHG for efficient cell cycle reentry. RNA. 2020; 26:1603–1620. 32675111 10.1261/rna.075309.120 PMC7566567

[22] Pawlicki J.M. , Steitz J.A. Primary microRNA transcript retention at sites of transcription leads to enhanced microRNA production. J. Cell Biol. 2008; 182:61–76. 18625843 10.1083/jcb.200803111 PMC2447899

[23] Janas M.M. , Khaled M., Schubert S., Bernstein J.G., Golan D., Veguilla R.A., Fisher D.E., Shomron N., Levy C., Novina C.D. Feed-forward microprocessing and splicing activities at a microRNA-containing intron. PLoS Genet. 2011; 7:4–13. 10.1371/journal.pgen.1002330 PMC3197686 22028668

[24] Kim Y.K. , Kim V.N. Processing of intronic microRNAs. EMBO J. 2007; 26:775–783. 17255951 10.1038/sj.emboj.7601512 PMC1794378

[25] Sundaram G.M. , Common J.E.A., Gopal F.E., Srikanta S., Lakshman K., Lunny D.P., Lim T.C., Tanavde V., Lane E.B., Sampath P. ‘See-saw’ expression of microRNA-198 and FSTL1 from a single transcript in wound healing. Nature. 2013; 495:103–106. 23395958 10.1038/nature11890

[26] Melamed Z. , Levy A., Ashwal-Fluss R., Lev-Maor G., Mekahel K., Atias N., Gilad S., Sharan R., Levy C., Kadener S. et al. Alternative splicing regulates biogenesis of miRNAs located across exon–intron junctions. Mol. Cell. 2013; 50:869–881. 23747012 10.1016/j.molcel.2013.05.007

[27] Mattioli C. , Pianigiani G., Pagani F. A competitive regulatory mechanism discriminates between juxtaposed splice sites and pri-miRNA structures. Nucleic Acids Res. 2013; 41:8680–8691. 23863840 10.1093/nar/gkt614 PMC3794580

[28] Pianigiani G. , Licastro D., Fortugno P., Castiglia D., Petrovic I., Pagani F. Microprocessor-dependent processing of splice site overlapping microRNA exons does not result in changes in alternative splicing. RNA. 2018; 24:1158–1171. 29895677 10.1261/rna.063438.117 PMC6097652

[29] Kopp F. , Mendell J.T. Review functional classification and experimental dissection of long noncoding RNAs. Cell. 2018; 172:393–407. 29373828 10.1016/j.cell.2018.01.011 PMC5978744

[30] Schlackow M. , Nojima T., Gomes T., Dhir A., Carmo-Fonseca M., Proudfoot N.J. Distinctive patterns of transcription and RNA processing for human lincRNAs. Mol. Cell. 2017; 65:25–38. 28017589 10.1016/j.molcel.2016.11.029 PMC5222723

[31] Nojima T. , Proudfoot N.J. Mechanisms of lncRNA biogenesis as revealed by nascent transcriptomics. Nat. Rev. Mol. Cell Biol. 2022; 23:389–406. 35079163 10.1038/s41580-021-00447-6

[32] Chang J. , Nicolas E., Marks D., Sander C., Lerro A., Buendia M.A., Xu C., Mason W.S., Moloshock T., Bort R. et al. miR-122, a mammalian liver-specific microRNA, is processed from hcr mRNA and may downregulate the high affinity cationic amino acid transporter CAT-1. RNA Biol. 2004; 1:106–113. 17179747 10.4161/rna.1.2.1066

[33] Dhir A. , Dhir S., Proudfoot N.J., Jopling C.L. Microprocessor mediates transcriptional termination of long noncoding RNA transcripts hosting microRNAs. Nat. Struct. Mol. Biol. 2015; 22:319–327. 25730776 10.1038/nsmb.2982 PMC4492989

[34] Jopling C.L. , Yi M., Lancaster A.M., Lemon S.M., Sarnow P. Modulation of hepatitis C virus RNA abundance by a liver-specific microRNA. Science. 2005; 309:1577–1581. 16141076 10.1126/science.1113329

[35] Hsu S. , Wang B., Kota J., Yu J., Costinean S., Kutay H., Yu L., Bai S., La Perle K., Chivukula R.R. et al. Essential metabolic, anti-inflammatory, and anti-tumorigenic functions of miR-122 in liver. J. Clin. Invest. 2012; 122:2871–2883. 22820288 10.1172/JCI63539 PMC3408748

[36] Kotake Y. , Sagane K., Owa T., Mimori-Kiyosue Y., Shimizu H., Uesugi M., Ishihama Y., Iwata M., Mizui Y. Splicing factor SF3b as a target of the antitumor natural product pladienolide. Nat. Chem. Biol. 2007; 3:570–575. 17643112 10.1038/nchembio.2007.16

[37] Yokoi A. , Kotake Y., Takahashi K., Kadowaki T., Matsumoto Y., Minoshima Y., Sugi N.H., Sagane K., Hamaguchi M., Iwata M. et al. Biological validation that SF3b is a target of the antitumor macrolide pladienolide. FEBS J. 2011; 278:4870–4880. 21981285 10.1111/j.1742-4658.2011.08387.x

[38] Reichholf B. , Herzog V.A., Fasching N., Manzenreither R.A., Sowemimo I., Ameres S.L. Time-resolved small RNA sequencing unravels the molecular principles of microRNA homeostasis. Mol. Cell. 2019; 75:756–768. 31350118 10.1016/j.molcel.2019.06.018 PMC6713562

[39] Wang R. , Simoneau C.R., Kulsuptrakul J., Bouhaddou M., Travisano K.A., Hayashi J.M., Carlson-Stevermer J., Zengel J.R., Richards C.M., Fozouni P. et al. Genetic screens identify host factors for SARS-CoV-2 and common cold coronaviruses. Cell. 2021; 184:106–119. 33333024 10.1016/j.cell.2020.12.004 PMC7723770

[40] Dye M.J. , Gromak N., Proudfoot N.J. Exon tethering in transcription by RNA polymerase II. Mol. Cell. 2006; 21:849–859. 16543153 10.1016/j.molcel.2006.01.032

[41] Gagliardi M. , Matarazzo M.R. Lanzuolo C. , Bodega B. RIP: RNA immunoprecipitation. Methods in Molecular Biology. 2016; 1480:NY Springer 73–86. 10.1007/978-1-4939-6380-5_7 27659976

[42] Duffy E.E. , Rutenberg-Schoenberg M., Stark C.D., Kitchen R.R., Gerstein M.B., Simon M.D. Tracking distinct RNA populations using efficient and reversible covalent chemistry. Mol. Cell. 2015; 59:858–866. 26340425 10.1016/j.molcel.2015.07.023 PMC4560836

[43] Rädle B. , Rutkowski A.J., Ruzsics Z., Friedel C.C., Koszinowski U.H., Dölken L. Metabolic labeling of newly transcribed RNA for high resolution gene expression profiling of RNA synthesis, processing and decay in cell culture. J. Vis. Exp. 2013; 10.3791/50195. PMC3854562 23963265

[44] Herzog V.A. , Reichholf B., Neumann T., Rescheneder P., Bhat P., Burkard T.R., Wlotzka W., von Haeseler A., Zuber J., Ameres S.L. Thiol-linked alkylation of RNA to assess expression dynamics. Nat. Methods. 2017; 14:1198–1204. 28945705 10.1038/nmeth.4435 PMC5712218

[45] Neumann T. , Herzog V.A., Muhar M., von Haeseler A., Zuber J., Ameres S.L., Rescheneder P. Quantification of experimentally induced nucleotide conversions in high-throughput sequencing datasets. BMC Bioinformatics. 2019; 20:258. 31109287 10.1186/s12859-019-2849-7 PMC6528199

[46] Ewels P.A. , Peltzer A., Fillinger S., Patel H., Alneberg J., Wilm A., Garcia M.U., Di Tommaso P., Nahnsen S. The nf-core framework for community-curated bioinformatics pipelines. Nat. Biotechnol. 2020; 38:276–278. 32055031 10.1038/s41587-020-0439-x

[47] Kozomara A. , Birgaoanu M., Griffiths-Jones S. miRBase: from microRNA sequences to function. Nucleic Acids Res. 2019; 47:D155–D162. 30423142 10.1093/nar/gky1141 PMC6323917

[48] Fromm B. , Høye E., Domanska D., Zhong X., Aparicio-Puerta E., Ovchinnikov V., Umu S.U., Chabot P.J., Kang W., Aslanzadeh M. et al. MirGeneDB 2.1: toward a complete sampling of all major animal phyla. Nucleic Acids Res. 2022; 50:D204–D210. 34850127 10.1093/nar/gkab1101 PMC8728216

[49] Hinske L.C. , França G.S., Torres H.A.M., Ohara D.T., Lopes-Ramos C.M., Heyn J., Reis L.F.L., Ohno-Machado L., Kreth S., Galante P.A.F. miRIAD—integrating microRNA inter- and intragenic data. Database. 2014; 2014:bau099. 25288656 10.1093/database/bau099 PMC4186326

[50] Cretu C. , Agrawal A.A., Cook A., Will C.L., Fekkes P., Smith P.G., Lührmann R., Larsen N., Buonamici S., Pena V. Structural basis of splicing modulation by antitumor macrolide compounds. Mol. Cell. 2018; 70:265–273. 29656923 10.1016/j.molcel.2018.03.011

[51] Nojima T. , Gomes T., Grosso A.R.F., Kimura H., Dye M.J., Dhir S., Carmo-Fonseca M., Proudfoot N.J. Mammalian NET-seq reveals genome-wide nascent transcription coupled to RNA processing. Cell. 2015; 161:526–540. 25910207 10.1016/j.cell.2015.03.027 PMC4410947

[52] Caizzi L. , Monteiro-Martins S., Schwalb B., Lysakovskaia K., Schmitzova J., Sawicka A., Chen Y., Lidschreiber M., Cramer P. Efficient RNA polymerase II pause release requires U2 snRNP function. Mol. Cell. 2021; 81:1920–1934. 33689748 10.1016/j.molcel.2021.02.016

[53] Kaida D. , Motoyoshi H., Tashiro E., Nojima T., Hagiwara M., Ishigami K., Watanabe H., Kitahara T., Yoshida T., Nakajima H. et al. Spliceostatin A targets SF3b and inhibits both splicing and nuclear retention of pre-mRNA. Nat. Chem. Biol. 2007; 3:576–583. 17643111 10.1038/nchembio.2007.18

[54] Valdmanis P.N. , Kim H.K., Chu K., Zhang F., Xu J., Munding E.M., Shen J., Kay M.A. miR-122 removal in the liver activates imprinted microRNAs and enables more effective microRNA-mediated gene repression. Nat. Commun. 2018; 9:5321. 30552326 10.1038/s41467-018-07786-7 PMC6294001

[55] Katoh T. , Sakaguchi Y., Miyauchi K., Suzuki T., Kashiwabara S., Baba T., Suzuki T. Selective stabilization of mammalian microRNAs by 3′ adenylation mediated by the cytoplasmic poly(A) polymerase GLD-2. Genes Dev. 2009; 23:433–438. 19240131 10.1101/gad.1761509 PMC2648654

[56] Bissels U. , Wild S., Tomiuk S., Holste A., Hafner M., Tuschl T., Bosio A. Absolute quantification of microRNAs by using a universal reference. RNA. 2009; 15:2375–2384. 19861428 10.1261/rna.1754109 PMC2779673

[57] Ribas J. , Ni X., Castanares M., Liu M.M., Esopi D., Yegnasubramanian S., Rodriguez R., Mendell J.T., Lupold S.E. A novel source for miR-21 expression through the alternative polyadenylation of VMP1 gene transcripts. Nucleic Acids Res. 2012; 40:6821–6833. 22505577 10.1093/nar/gks308 PMC3413119

[58] Fromm B. , Zhong X., Tarbier M., Friedländer M.R., Hackenberg M. The limits of human microRNA annotation have been met. RNA. 2022; 28:781–785. 35236776 10.1261/rna.079098.122 PMC9074900

[59] Seńis E. , Mockenhaupt S., Rupp D., Bauer T., Paramasivam N., Knapp B., Gronych J., Grosse S., Windisch M.P., Schmidt F. et al. TALEN/CRISPR-mediated engineering of a promoterless anti-viral RNAi hairpin into an endogenous miRNA locus. Nucleic Acids Res. 2017; 45:e3. 27614072 10.1093/nar/gkw805 PMC5224498

[60] Krol J. , Busskamp V., Markiewicz I., Stadler M.B., Ribi S., Richter J., Duebel J., Bicker S., Fehling H.J., Schübeler D. et al. Characterizing light-regulated retinal microRNAs reveals rapid turnover as a common property of neuronal microRNAs. Cell. 2010; 141:618–631. 20478254 10.1016/j.cell.2010.03.039

[61] Han J. , Mendell J.T. MicroRNA turnover: a tale of tailing, trimming, and targets. Trends Biochem. Sci. 2023; 48:26–39. 35811249 10.1016/j.tibs.2022.06.005 PMC9789169

[62] Kingston E.R. , Bartel D.P. Global analyses of the dynamics of mammalian microRNA metabolism. Genome Res. 2019; 29:1777–1790. 31519739 10.1101/gr.251421.119 PMC6836734

[63] Kim H. , Kim J., Kim K., Chang H., You K., Kim V.N. Bias-minimized quantification of microRNA reveals widespread alternative processing and 3′ end modification. Nucleic Acids Res. 2019; 47:2630–2640. 30605524 10.1093/nar/gky1293 PMC6411932

[64] Randall G. , Panis M., Cooper J.D., Tellinghuisen T.L., Sukhodolets K.E., Pfeffer S., Landthaler M., Landgraf P., Kan S., Lindenbach B.D. et al. Cellular cofactors affecting hepatitis C virus infection and replication. Proc. Natl Acad. Sci. U.S.A. 2007; 104:12884–12889. 17616579 10.1073/pnas.0704894104 PMC1937561

[65] Luna J.M. , Barajas J.M., Teng K., Sun H.-L., Moore M.J., Rice C.M., Darnell R.B., Ghoshal K. Argonaute CLIP defines a deregulated miR-122-bound transcriptome that correlates with patient survival in human liver cancer. Mol. Cell. 2017; 67:400–410. 28735896 10.1016/j.molcel.2017.06.025 PMC5603316

[66] Kirstein N. , Dokaneheifard S., Cingaram P.R., Valencia M.G., Beckedorff F., Gomes Dos Santos H., Blumenthal E., Tayari M.M., Gaidosh G.S., Shiekhatter R The integrator complex regulates microRNA abundance through RISC loading. Sci. Adv. 2023; 9:eadf0597. 36763664 10.1126/sciadv.adf0597 PMC9916992

[67] Aslan D. , Garde C., Nygaard M.K., Helbo A.S., Dimopoulos K., Hansen J.W., Severinsen M.T., Treppendahl M.B., Sjø L.D., Grønbæk K. et al. Tumor suppressor microRNAs are downregulated in myelodysplastic syndrome with spliceosome mutations. OncoTargets Ther. 2016; 7:9951–9963. 10.18632/oncotarget.7127 PMC4891095 26848861

[68] Du P. , Wang L., Sliz P., Gregory R.I. A biogenesis step upstream of Microprocessor controls miR-17∼92 expression. Cell. 2015; 162:885–899. 26255770 10.1016/j.cell.2015.07.008 PMC4537828

[69] Kurimoto R. , Chiba T., Ito Y., Matsushima T., Yano Y., Miyata K., Yashiro Y., Suzuki T., Tomita K., Asahara H. The tRNA pseudouridine synthase TruB1 regulates the maturation of let-7 miRNA. EMBO J. 2020; 39:e104708. 32926445 10.15252/embj.2020104708 PMC7560213

[70] Jia T. , Zhang B., You C., Zhang Y., Zeng L., Li S., Johnson K.C.M., Yu B., Li X., Chen X. The *Arabidopsis* MOS4-associated complex promotes microRNA biogenesis and precursor messenger RNA splicing. Plant Cell. 2017; 29:2626–2643. 28947490 10.1105/tpc.17.00370 PMC5774577

[71] Li S. , Xu R., Li A., Liu K., Gu L., Li M., Zhang H., Zhang Y., Zhuang S., Wang Q. et al. SMA1, a homolog of the splicing factor Prp28, has a multifaceted role in miRNA biogenesis in *Arabidopsis*. Nucleic Acids Res. 2018; 46:9148–9159. 29982637 10.1093/nar/gky591 PMC6158494

[72] Liang C. , Cai Q., Wang F., Li S., You C., Xu C., Gao L., Cao D., Lan T., Zhang B. et al. Arabidopsis RBV is a conserved WD40 repeat protein that promotes microRNA biogenesis and ARGONAUTE1 loading. Nat. Commun. 2022; 13:1217. 35260568 10.1038/s41467-022-28872-x PMC8904849

[73] Convertini P. , Shen M., Potter P.M., Palacios G., Lagisetti C., De La Grange P., Horbinski C., Fondufe-Mittendorf Y.N., Webb T.R., Stamm S. Sudemycin E influences alternative splicing and changes chromatin modifications. Nucleic Acids Res. 2014; 42:4947–4961. 24623796 10.1093/nar/gku151 PMC4005683

[74] Bonnal S. , Vigevani L., Valcárcel J. The spliceosome as a target of novel antitumour drugs. Nat. Rev. Drug Discov. 2012; 11:847–859. 23123942 10.1038/nrd3823

[75] Salton M. , Misteli T. Small molecule modulators of pre-mRNA splicing in cancer therapy. Trends Mol. Med. 2016; 22:28–37. 26700537 10.1016/j.molmed.2015.11.005 PMC4707101

[76] Desterro J. , Bak-Gordon P., Carmo-Fonseca M. Targeting mRNA processing as an anticancer strategy. Nat. Rev. Drug Discov. 2020; 19:112–129. 31554928 10.1038/s41573-019-0042-3

[77] Slack F.J. , Chinnaiyan A.M. The role of non-coding RNAs in oncology. Cell. 2019; 179:1033–1055. 31730848 10.1016/j.cell.2019.10.017 PMC7347159

[78] Denzler R. , Agarwal V., Stefano J., Bartel D.P., Stoffel M. Assessing the ceRNA hypothesis with quantitative measurements of miRNA and target abundance. Mol. Cell. 2014; 54:766–776. 24793693 10.1016/j.molcel.2014.03.045 PMC4267251

[79] Janssen H.L.A. , Reesink H.W., Lawitz E.J., Zeuzem S., Rodriguez-Torres M., Patel K., van der Meer A.J., Patick A.K., Chen A., Zhou Y. et al. Treatment of HCV infection by targeting microRNA. N. Engl. J. Med. 2013; 368:1685–1694. 23534542 10.1056/NEJMoa1209026

[80] van der Ree M.H. , de Vree J.M., Stelma F., Willemse S., van der Valk M., Rietdijk S., Molenkamp R., Schinkel J., van Nuenen A.C., Beuers U. et al. Safety, tolerability, and antiviral effect of RG-101 in patients with chronic hepatitis C: a phase 1B, double-blind, randomised controlled trial. Lancet. 2017; 389:709–717. 28087069 10.1016/S0140-6736(16)31715-9

[81] Esau C. , Davis S., Murray S.F., Yu X.X., Pandey S.K., Pear M., Watts L., Booten S.L., Graham M., McKay R. et al. miR-122 regulation of lipid metabolism revealed by *in vivo* antisense targeting. Cell Metab. 2006; 3:87–98. 16459310 10.1016/j.cmet.2006.01.005

[82] Krützfeldt J. , Rajewsky N., Braich R., Rajeev K.G., Tuschl T., Manoharan M., Stoffel M. Silencing of microRNAs *in vivo* with “antagomirs”. Nature. 2005; 438:685–689. 16258535 10.1038/nature04303

[83] Amen A.M. , Loughran R.M., Huang C.-H., Lew R.J., Ravi A., Guan Y., Schatoff E.M., Dow L.E., Emerling B.M., Fellmann C. Endogenous spacing enables co-processing of microRNAs and efficient combinatorial RNAi. Cell Rep. Methods. 2022; 2:100239. 35880017 10.1016/j.crmeth.2022.100239 PMC9308131

