# Supplementary material for: MicroRNA biogenesis is broadly disrupted by inhibition of the splicing factor SF3B1
Source: Nucleic Acids Res. 2024 Jun 17;52(15):9210–29. doi: 10.1093/nar/gkae505 (PMC11347158; doi:10.1093/nar/gkae505)
Supplement: gkae505_Supplemental_Files [file gkae505_supplemental_files.zip › Revised Downie et al Supplementary NAR-03012.pdf]

Supplementary Table 1: Antisense oligonucleotides

Antisense oligonucleotides were fully modified with 2' methyoxyethyl bases and a phosphorothioate backbone.

ASO name	ASO sequence (5' to 3')
Scr	ACAGUCCUAGUGGCUAGAUACCGC
3'SS	GGCTTTGAATAATCTGCAAAATAC
5'SS	GCCGCACTTACCCCCAGTCAGCTC

Supplementary Table 2: Primer sequences

Primer name	Primer sequence (5' to 3')	Use
Pri-122 intron qF 1	GGGATCCTCCTGCCTAAGAC	RT-qPCR to detect unspliced pri-miR-122 (Figure 1, S1)
Pri-122 intron qR 1	GAAAAGGCCTTGAGGGATTC	
Pri-122 intron qF 2	AGGGAGATCTCAGGAAGTAAGG	RT-qPCR to detect unspliced pri-miR-122 (Figure 2)
Pri-122 intron qR 2	GGCCTTGAGGGATTCTGTTTAG	
Pri-122 spliced qF 1	CTGACTGGGGATTATTCAAAGC	RT-qPCR to detect spliced pri-miR-122 (Figure 1)
Pri-122 spliced qR 1	TCGCTGACCATCCACAAGAG	
Pri-122 spliced qF 2	GAAGGGCCTTGGACTGAGA	RT-qPCR to detect spliced pri-miR-122 (Figure S1)
Pri-122 spliced qR 2	GCTTGGAGCGCTCTATCCT	
Pri-122 spliced qF 3	AGGGCCTTGGACTGAGA	RT-qPCR to detect spliced pri-miR-122 (Figure 2, 6)
Pri-122 spliced qR 3	CGCTGACCATCCACAAGAG	
Pri-122 exon2 qF	CTCTCTCTCCATCTTCCTCTCT	RT-qPCR to detect exon 2 of pri-miR-122 (Figure 2)
Pri-122 exon 2 qR	CTACTCTCCGTGGCATCTATTG	
Pri-122 unspliced qF	CTTCCATCTGTTCCTTCCCTTT	RT-qPCR to detect unspliced pri-miR-122 (Figure 6)
Pri-122 unspliced qR	AGAAACAGTGAGAGGTGAACAA	
Pri-122 Drosha qF	TTCTCTGCTTAGGTCACAAT	RT-qPCR to detect Drosha processing of pri-miR-122 (Figure 7)
Pri-122 Drosha qF	AACACCATTGTCACACTCCA	
Pri-122 WT qF	ATGGAGAAGTGGAGGATGCTCA	RT-qPCR specific to WT pri-miR-122 (Figure 6)
Pri-122 WT qR	ATCGCCACCTCCTGGTCATA	
Pri-122 3'SSΔ qF	CTGGGCCATAGAAATTGAGG	RT-qPCR specific to 3'SSΔ pri-miR-122 (Figure 6)
Pri-122 3'SSΔ qR	CAGGGCTCCATAGAATCAACCT	
Pri-17 qF	GGAAGCCAGAAGAGGAGGAA	RT-qPCR to detect pri-miR-17~92a
Pri-17 qR	AAGTGGTGGCTCTTCCAATG	
Pri-23b qF	GCCTGGAGAGAAAGGGATCTT	RT-qPCR to detect pri-miR-23b~27b~24-1
Pri-23b qR	AGGGACTCCAGAGATGACCA	
Pri-29 qF	ATGGAGCACTTGCTTGCTTT	RT-qPCR to detect pri-miR-29a~29b-1
Pri-29 qR	GTCACTCTTGGGTGGGAAGA	

Pri-122 RIP qF	CTTGTACCCGTGATGCTTCTT	RT-qPCR for pri-miR-122 in RIP (Figure 6)
Pri-122 RIP qR	CACCTCCACATTCCACCATT	
Actin qF	AGCACAGAGCCTCGCCTTT	RT-qPCR control (Figures 1, S1)
Actin qR	TCATCATCCATGGTGAGCTG	
18S qF	CAGCCACCCGAGATTGAGCA	RT-qPCR control (Figures 2-6)
18S qR	TAGTAGCGACGGGCGGTGTG	
3'SS gDNA F	CAATGCCACTATGAGCAACC	CRISPR screening
3'SS gDNA R	AGCTCTGCTGGGAAGAAACA	

Supplementary Table 3: Northern probes
All small RNA northern probes were unmodified DNA.

Target	Oligonucleotide probe sequence (5' to 3')
miR-122-5p	ACAAACACCATTGTCACACTCCA
miR-122-3p	TTTAGTGTGATAATGGCGTTTGA
miR-17-5p	CTACCTGCACTGTAAGCACTTTG
miR-21-5p	TCAACATCAGTCTGATAAGCTA
miR-21-3p	ACAGCCCATCGACTGGTGTTG
U6 snRNA	ATATGGAACGCTTCACGAATT

Supplementary Table 4: SLAMseq data
Data table shows CPM and T>C conversion rate for *Control* (no 4SU, DMSO), *DMSO* (4SU, DMSO) and *PlaB* (4SU, PlaB) conditions in each of four datasets prepared following independent experiments (A-D). Annotation of features of miRNA genes, as shown in Figure 5, is included.

SUPPLEMENTARY METHODS

Spliceostatin A

Spliceostatin A (SSA) was a gift of Minoru Yoshida (RIKEN, Japan) and was dissolved in methanol and stored at -20°C. It was applied to cells at 100 ng/ml final concentration. The same volume of methanol was applied to cells as a control. Total RNA was harvested from cells using TRI Reagent at 8 h post treatment.

SF3B1 knockdown

Huh7 cells cultured in 6 well plates were transfected with an siRNA targeting SF3B1 (Dharmacon ON-TARGETplus, J-020061-13-0005) or a control siRNA (Dharmacon ON-TARGETplus Non-Targeting Control #3) at 10 nM final concentration using Lipofectamine RNAiMax (Invitrogen) according to the manufacturer's instructions. Cells were cultured for 24 h, expanded into 10 cm plates, and 24 h later transfected with a second hit of siRNA and transfected for a further 24 h before chromatin isolation was carried out. Total protein was extracted from cells treated in parallel for western blot.

Cell viability assays

Cell viability following PlaB or 4SU treatment was assessed using alamarBlue reagent (ThermoFisher), according to the manufacturer's instructions. To assess the effects of PlaB on cell viability, Huh7 cells were plated in 96 well plates, then treated with 1 μM PlaB or an equivalent volume of DMSO for 4 h or 24 h. Following this, the alamarBlue reagent was added for an additional 4 h. Absorbance was then measured using a plate reader (Biotek). To assess the effects of 4SU, Huh7 cells were treated with concentrations of 4SU ranging from 250-5000 μM for 4 h or 24 h. All alamarBlue assays were performed in triplicate wells, with untreated cells included for normalization.

Western blotting

Equal volumes of chromatin, nucleoplasmic and cytoplasmic fractions from Huh7 cells +/- PlaB treatment were added to SDS PAGE loading buffer to 1 X final concentration and separated by 10% tris-glycine SDS–PAGE. For SF3B1 western, total protein was isolated as described previously (33). 100%, 50% and 25% molecular weight of protein from Control siRNA-transfected cells were run in parallel to 100% from SF3B1 siRNA-transfected cells to allow estimation of level of knockdown. Proteins were transferred to nitrocellulose (0.45 μM, Amersham Biosciences) by semi-dry blotting and detection was carried out with standard western blotting techniques. Primary antibodies were Histone 3 (Proteintech 17168-1-AP), β-tubulin (Abcam ab6046) and SF3B1 (Proteintech 27684-1-AP), and HRP-conjugated anti-rabbit secondary antibody was A6154 (Sigma). Signals were detected with Pierce ECL substrate (ThermoFisher) and visualized on a LAS-3000 Imager (FujiFilm).

Supplementary Figure 1

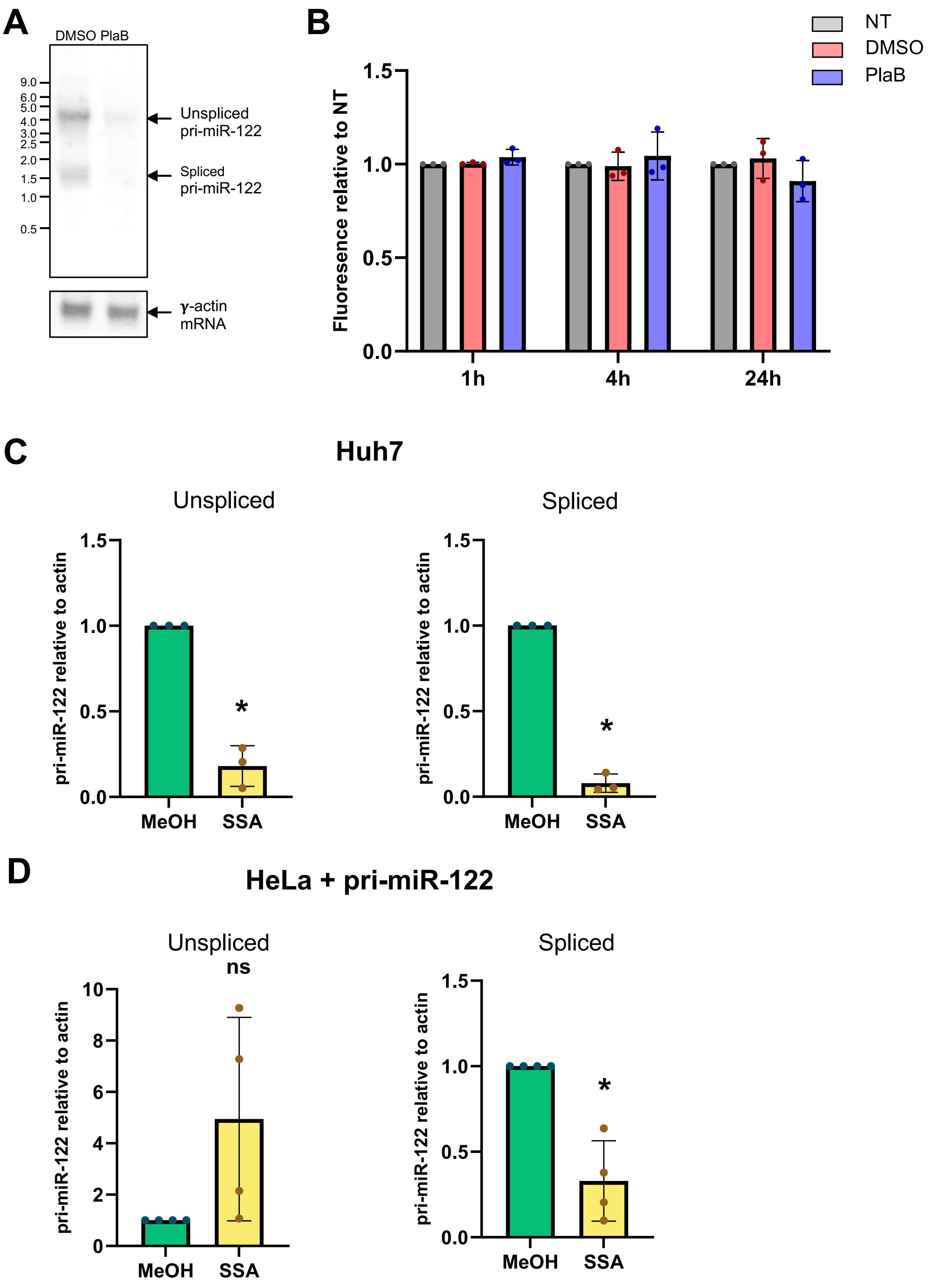

Supplementary Figure 1. Effects of SF3B1 inhibition on pri-miR-122

(A) Total RNA extracted from Huh7 cells treated with 1 μM PlaB for 4 h was analysed by northern blot using a radiolabelled probe specific to exon 2 of pri-miR-122. Unspliced and spliced pri-miR-122 were both reduced by PlaB treatment. The blot was re-probed for γ-actin mRNA as a loading control. (B) Huh7 cells treated with 1 μM PlaB for either 4 h or 24 h were then treated with alamarBlue reagent for 4 h, and absorbance was measured to assess cell viability. Absorbance from PlaB-treated cells or DMSO-treated control cells was normalized to that of non-treated cells (NT). (C) Huh7 cells were treated with the SF3B1 inhibitor SSA (100 ng/ml for 8 h) and total RNA isolated. Unspliced or spliced pri-miR-122 was measured by RT-qPCR, and data normalized to actin mRNA and shown relative to methanol-treated control. (D) HeLa cells were transfected with a pri-miR-122 plasmid and pTAT for 48 h before treatment with SSA for a further 8 h. Unspliced and spliced pri-miR-122 measured by RT-qPCR were normalized to actin mRNA and shown relative to methanol-treated control. Northern blot is representative of two independent experiments. All other data represent mean of at least three independent experiments, with error bars showing SD. *$p<0.05$. n.s. not significant.

Supplementary Figure 2

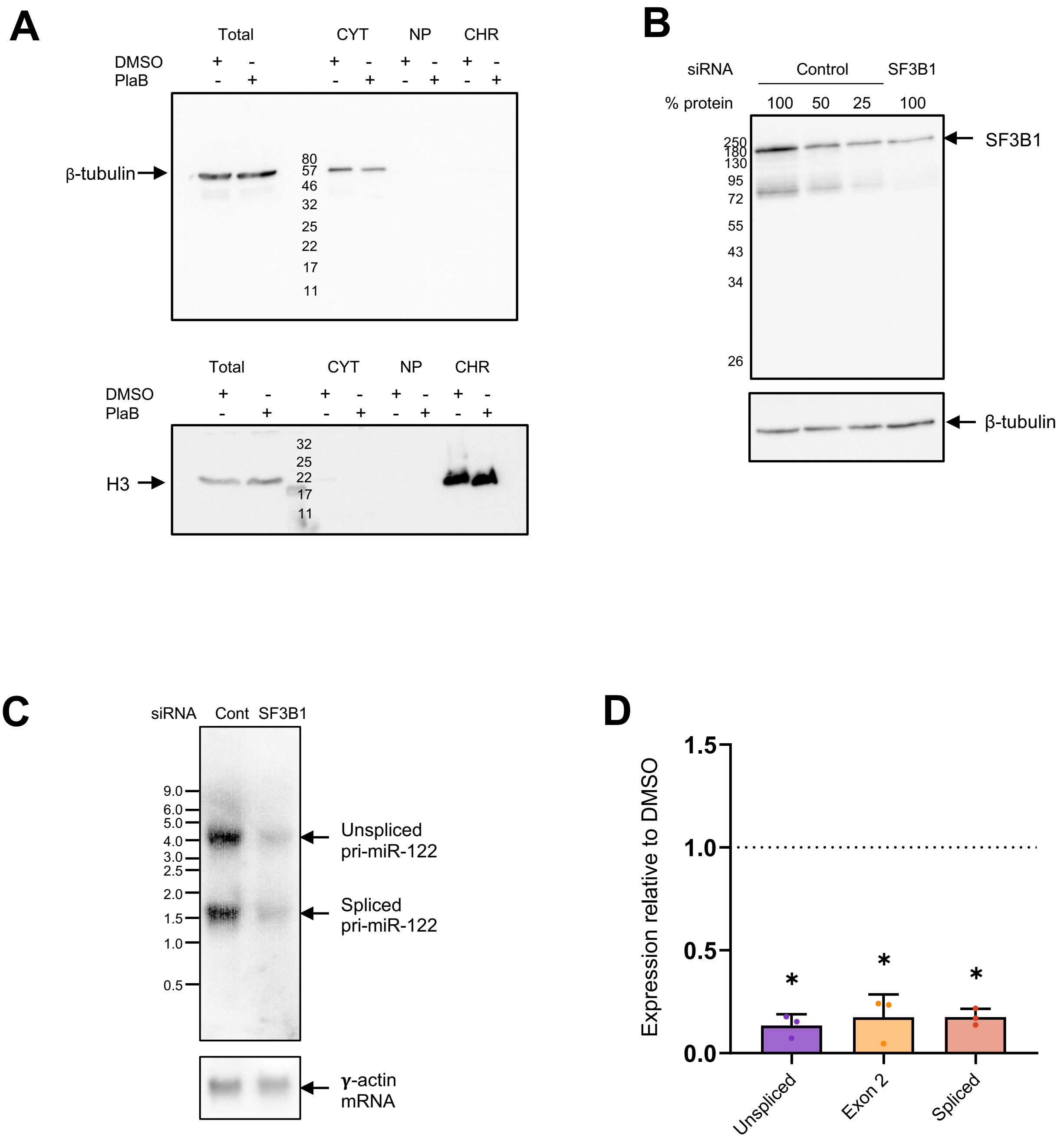

Supplementary Figure 2. Biochemical isolation of chromatin and effects of SF3B1 knockdown on pri-miR-122.
(A) Chromatin, nucleoplasm and cytoplasm were biochemically separated from Huh7 cells treated with 1 μM PlaB for 4 h or equivalent volume of DMSO. In parallel to RNA isolation from the chromatin fraction, protein was isolated from each fraction and separated by western blot, with total cell lysates from DMSO or PlaB-treated Huh7 cells for comparison. Blots were probed with antibodies specific to Histone 3 (H3; chromatin) and β-tubulin (cytoplasm) to confirm effective fractionation. CYT=cytoplasm, NP=nucleoplasm, CHR=chromatin. (B) Western blot showing siRNA-mediated knockdown of SF3B1 in Huh7 cells relative to different concentrations of total protein from control siRNA-treated cells. (C) Northern blot showing reduction of unspliced and spliced pri-miR-122 in total RNA from Huh7 cells following SF3B1 knockdown. γ-actin mRNA was used as a loading control. (D) Chromatin-associated RNA was isolated from Huh7 cells transfected with SF3B1 siRNA or a non-targeting control. Exon 2 (unspliced and spliced), unspliced and spliced pri-miR-122 were measured by RT-qPCR, normalized to 18S rRNA, and shown relative to the non-targeting siRNA control (dotted line at 1). Northern blot is representative of two independent experiments and all other data are representative of three independent experiments. *p<0.05

Supplementary Figure 3

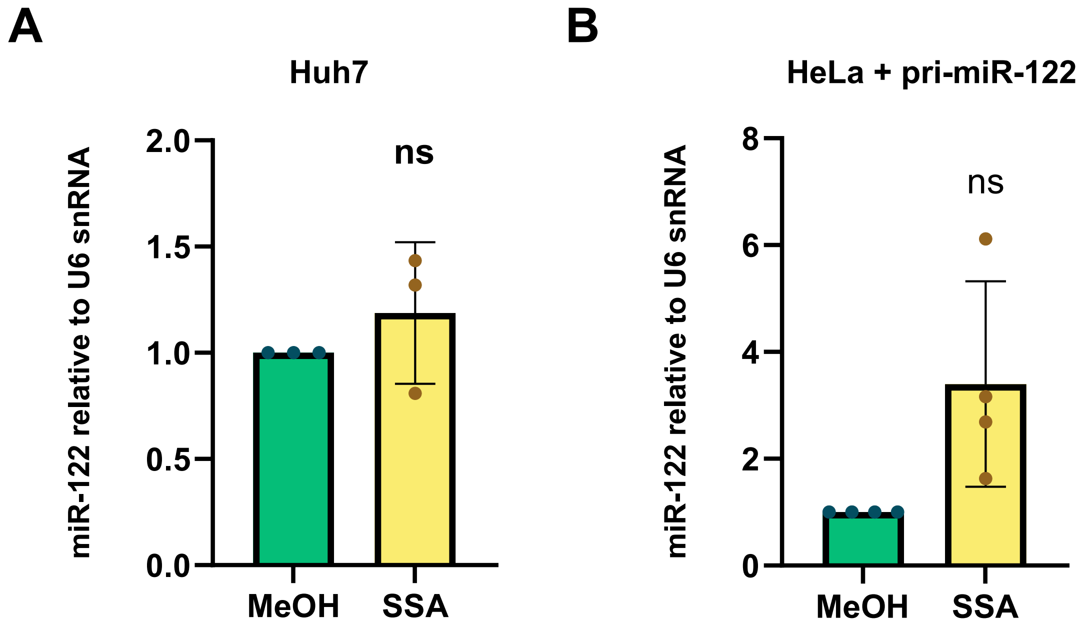

Supplementary Figure 3. Mature miR-122 is unaffected by SSA.
(A) Huh7 cells were treated with the SF3B1 inhibitor SSA (100 ng/ml for 8 h) and total RNA isolated. Mature miR-122 was analyzed by RT-qPCR, normalized to U6 snRNA, and is shown relative to methanol-treated control. (B) As (A), except that HeLa cells transfected with pHIV-LTR-pri-miR-122 were used. Data represent mean of at least three independent experiments, with error bars showing SD. n.s. not significant.

Supplementary Figure 4

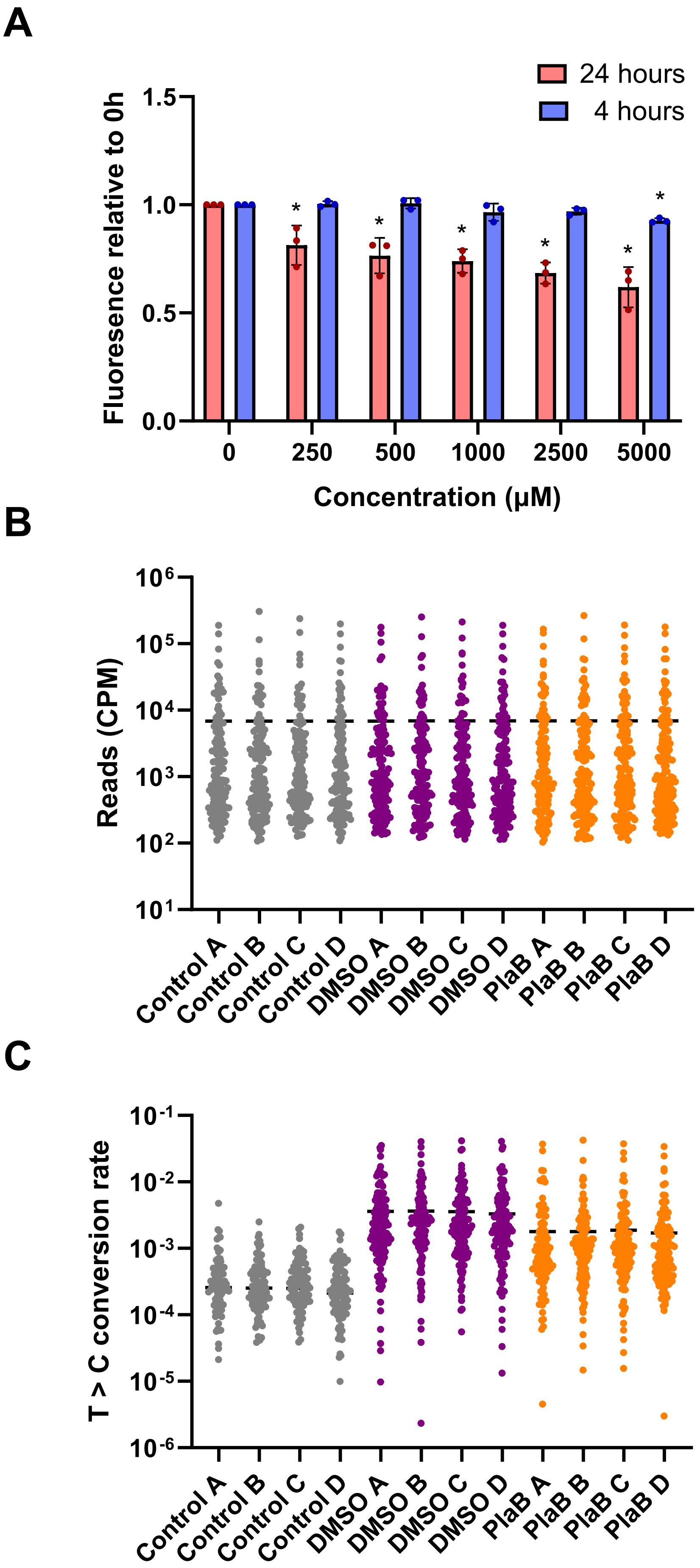

Supplementary Figure 4. Huh7 cell viability is unaffected by 4SU under SLAMseq conditions, and SLAMseq datasets are reproducible.

(A) Huh7 cells treated with different concentrations of 4SU for either 4 h or 24 h were then treated with alamarBlue reagent for 4 h, and absorbance was measured to assess cell viability. Absorbance from 4SU-treated cells was normalized to untreated cells. Data represent mean of three independent experiments, with error bars showing SD. *p<0.05. (B) Read counts of miRNAs in SLAMseq datasets from *Control* (DMSO, no 4SU), *DMSO* (DMSO+4SU) and *PlaB* (PlaB+4SU) conditions, shown as CPM for all miRNAs above the 100 CPM threshold. Data for four independent experiments (A-D) are shown separately. (C) As (B), except that T>C conversion rates are shown. Note that miRNAs for which T>C is 0 could not be plotted due to the log scale so were excluded. This includes 357 miRNAs across the 12 datasets (205 Control, 47 DMSO, 105 PlaB).

Supplementary Figure 5

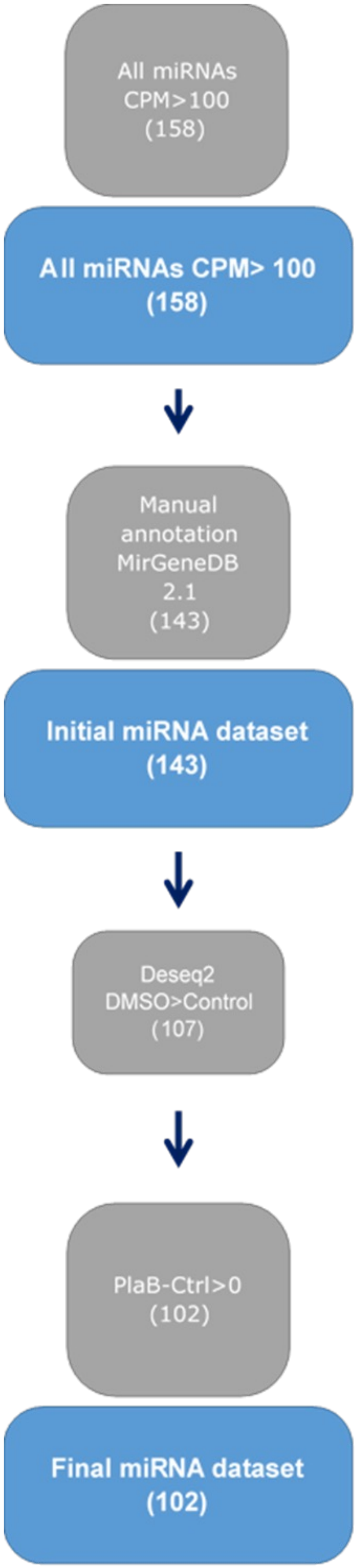

Supplementary Figure 5. Filtering of SLAMseq miRNA dataset.
Flowchart depicting filtering steps for miRNAs in SLAMseq data. Grey squares indicate filtering steps and blue squares indicate datasets referenced in the text. Numbers in parentheses refer to the remaining number of miRNAs after the relevant filters were applied.

Supplementary Figure 6

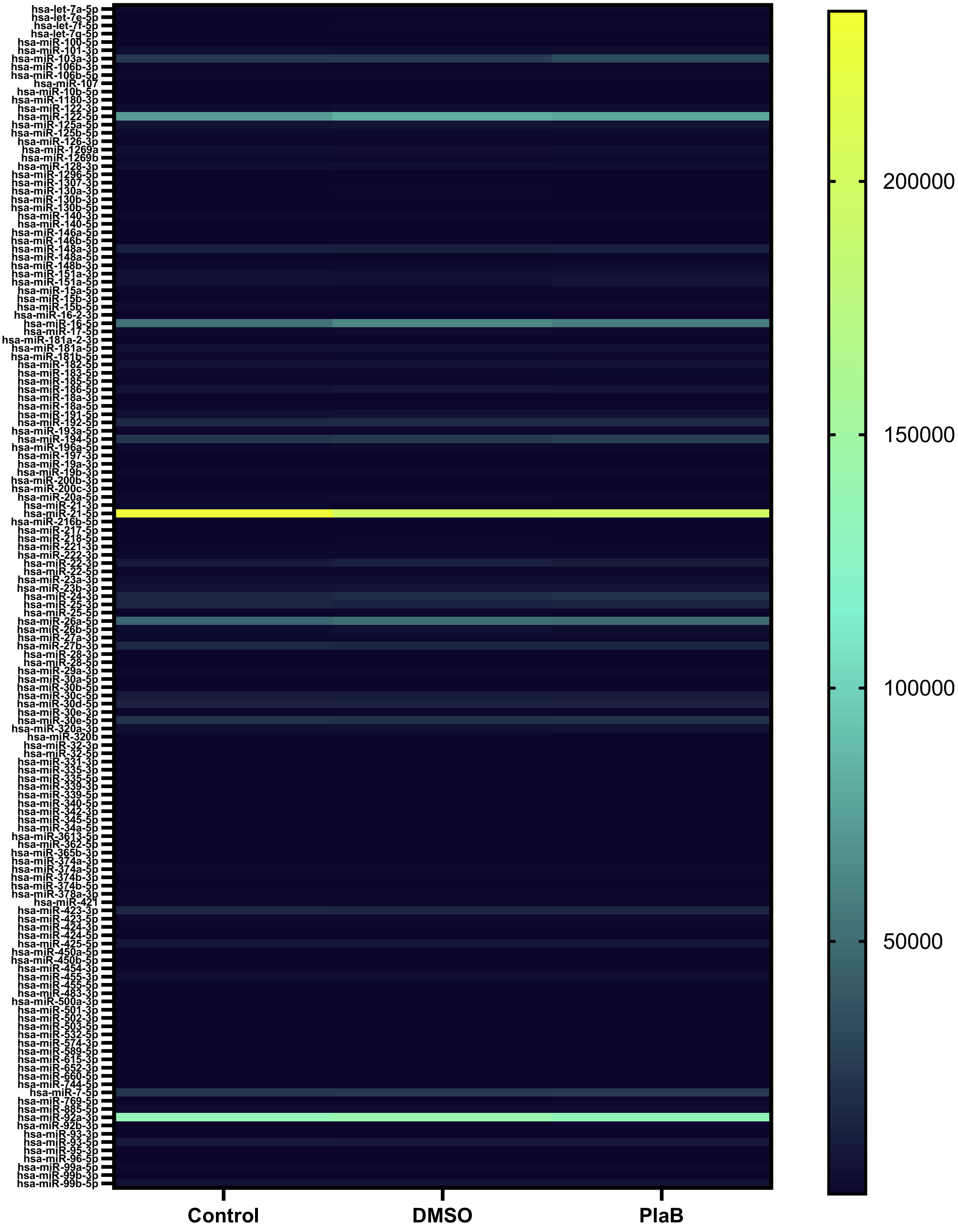

Supplementary Figure 6. Read counts in SLAMseq data.
(A) Heatmap showing read counts of miRNAs in SLAMseq datasets from *Control* (DMSO, no 4SU), *DMSO* (DMSO+4SU) and *PlaB* (PlaB+4SU) conditions, shown as CPM for all miRNAs in the 'Initial miRNA Dataset'. Data represent mean of the four independent experiments, A-D.

Supplementary Figure 7

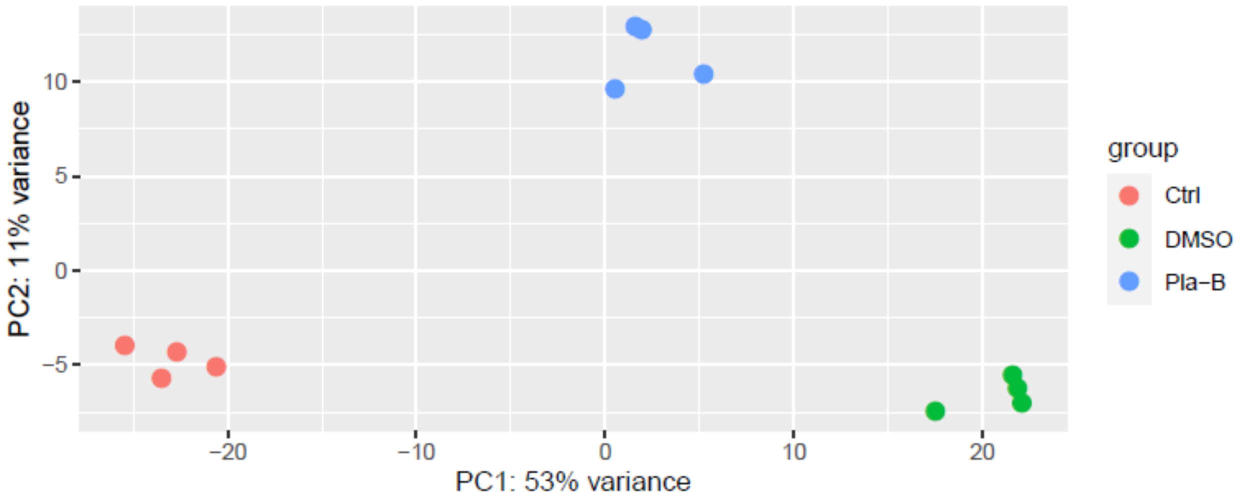

Supplementary Figure 7. Principal component analysis (PCA) of T>C conversion rate. PCA generated from DEseq2 comparison of T>C conversion rate in *PlaB*, *DMSO* and *Control* datasets for experiments A-D.

Supplementary Figure 8

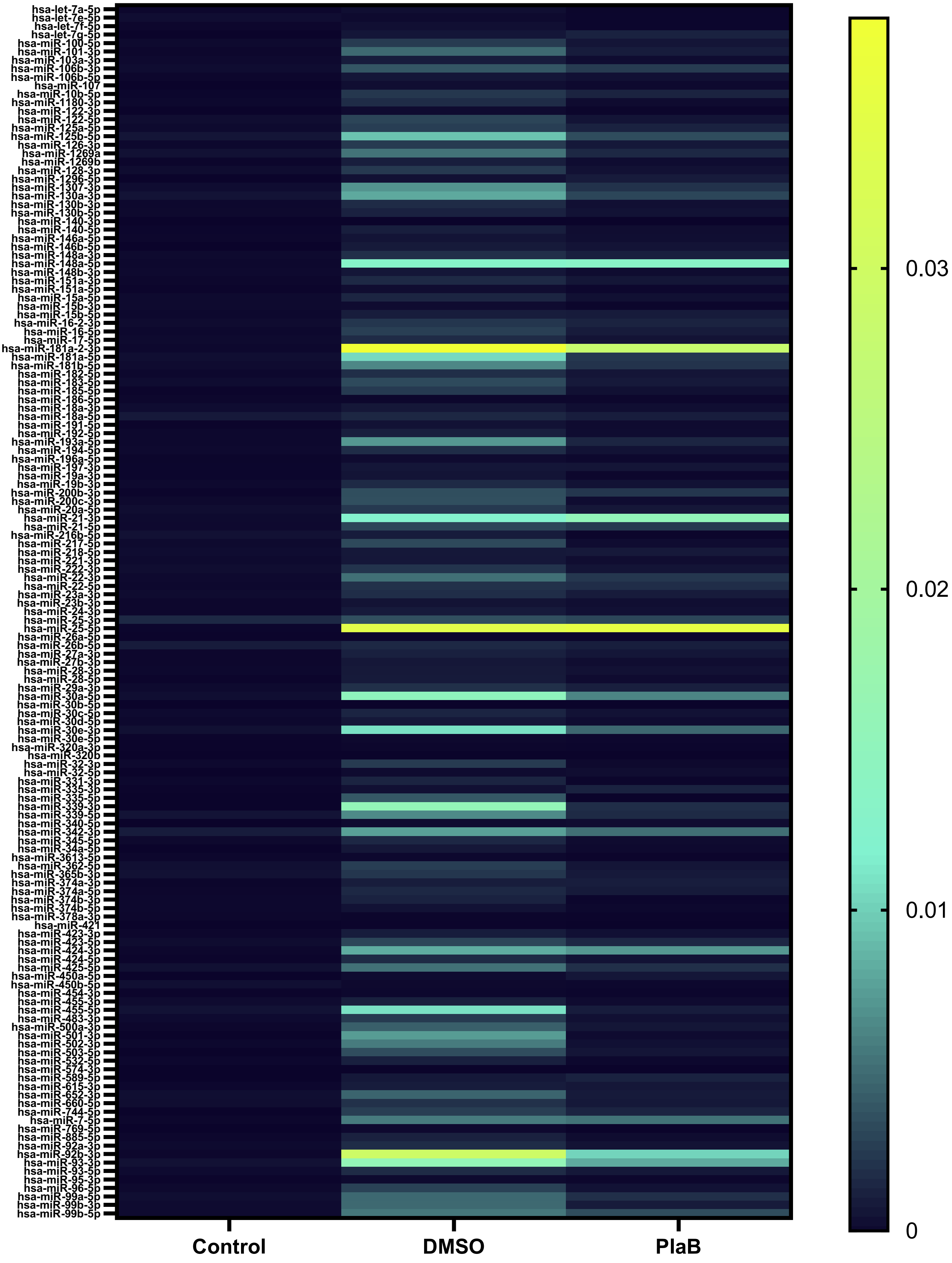

Supplementary Figure 8. T>C conversion rates in SLAMseq data.
(A) Heatmap showing T>C conversion rates in SLAMseq datasets from *Ctrl* (DMSO, no 4SU), *DMSO* (DMSO+4SU) and *PlaB* (PlaB+4SU) conditions, shown for all miRNAs above the 100 CPM threshold. Data represent mean of the four independent experiments, A-D.

Supplementary Figure 9

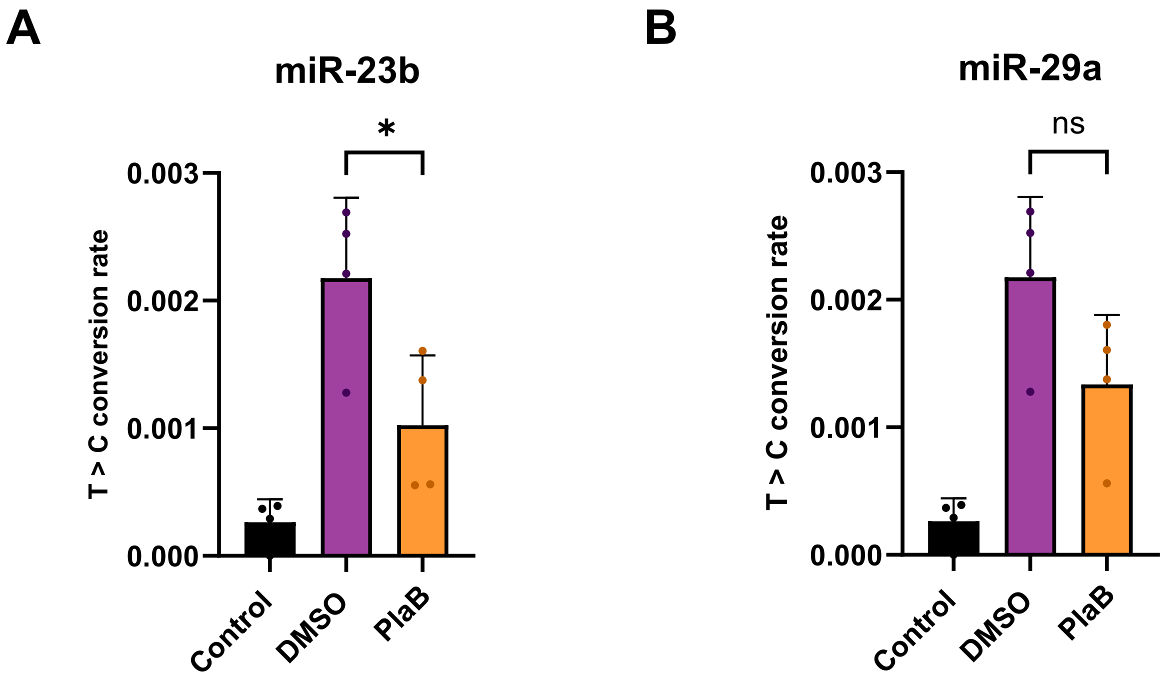

Supplementary Figure 9. Effect of PlaB on synthesis of miR-23b-3p and miR-29a-3p measured by SLAMseq.

T>C conversion rates are shown for (A) miR-23b-3p and (B) miR-29a-3p in *Control, DMSO and PlaB* conditions. Data represent mean of four independent experiments, with error bars showing SD. *p<0.05. n.s. not significant.

Supplementary Figure 10

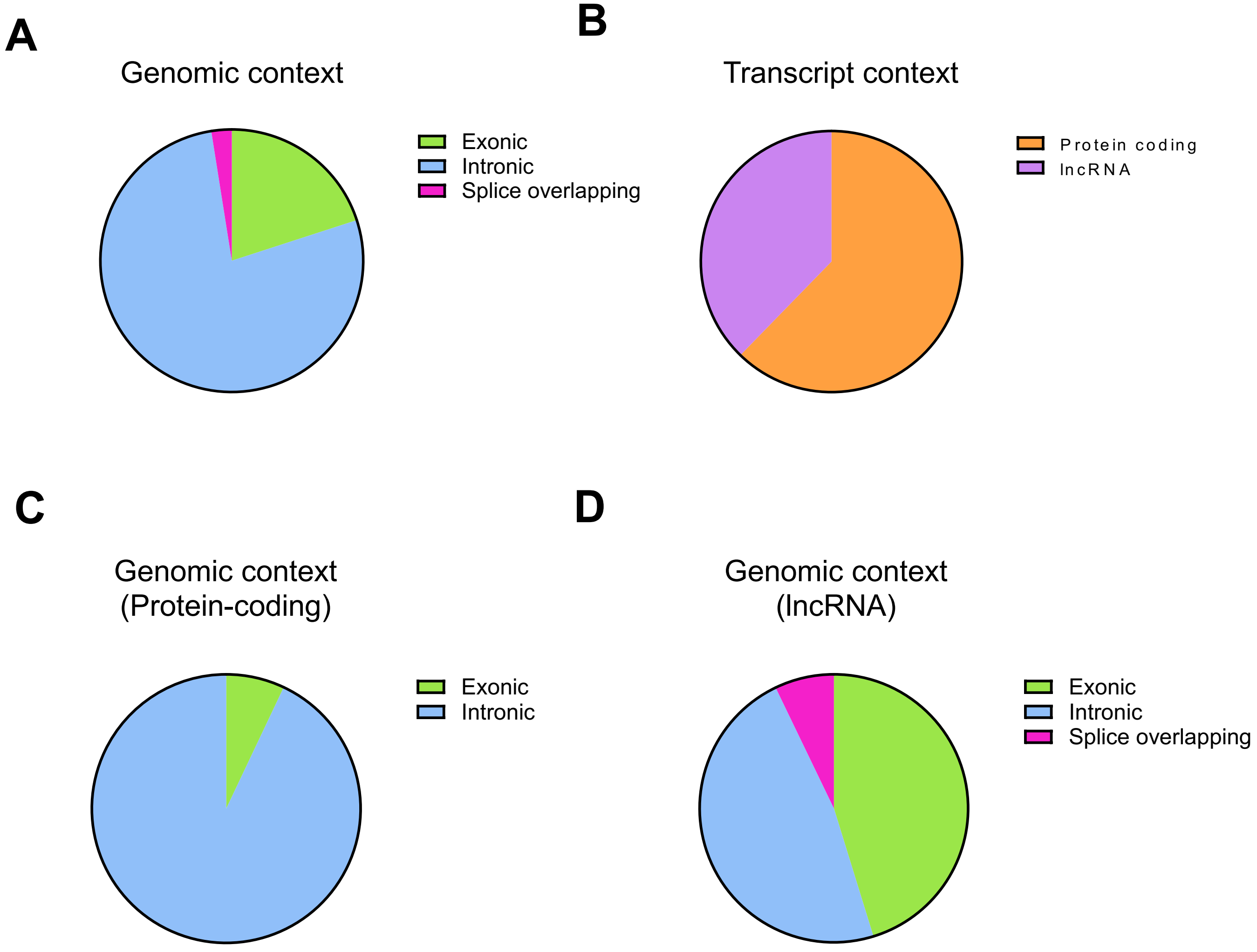

Supplementary Figure 10. Genomic distribution of miRNAs.
Pie charts showing distribution of 143 manually annotated miRNAs with expression >100 CPM in SLAMseq datasets (Initial miRNA Dataset) between (A) exonic, intronic or splice overlapping locations or (B) lncRNA versus protein coding gene location. (C) miRNAs located in protein coding genes were classified by exonic or intronic location. (D) miRNAs located in lncRNA genes were classified by exonic, intronic or splice overlapping location.

Supplementary Figure 11

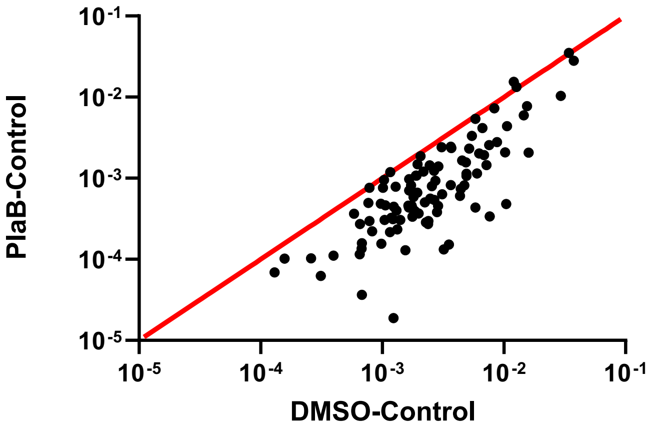

Supplementary Figure 11. PlaB reduces synthesis of most miRNAs.
Scatterplot comparing the effects of PlaB and DMSO on individual miRNAs, following background subtraction (PlaB-Control compared to DMSO-Control). Data represent mean of four independent experiments, A-D. Red identity line drawn at x=y.

Supplementary Figure 12

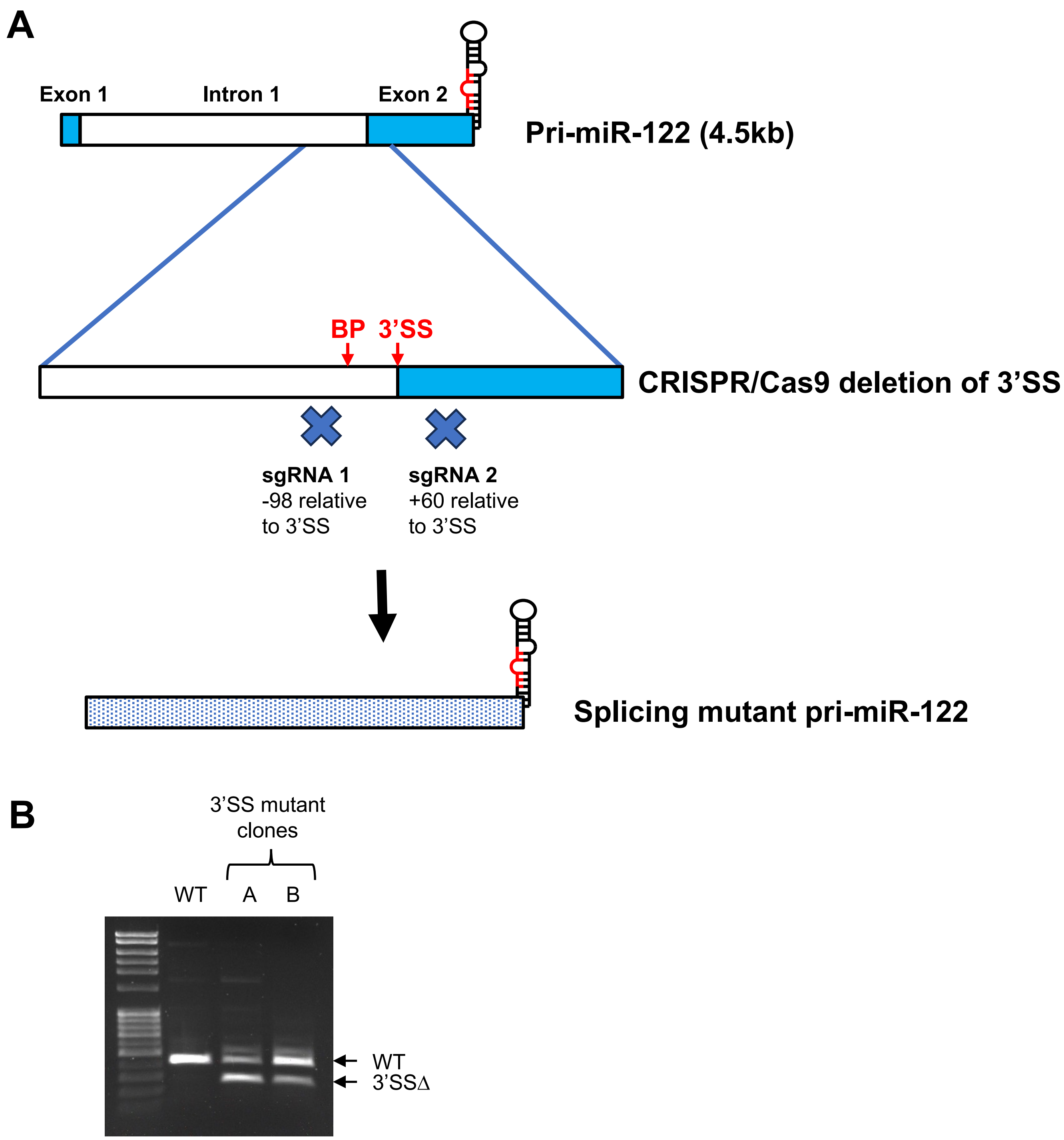

Supplementary Figure 12. Generation of heterozygous 3’SSΔ Huh7 cell lines by CRISPR/Cas9 genome modification.

(A) Design of the CRISPR/Cas9 approach using two sgRNAs, one targeting either side of the branch point-polypyrimidine tract-3’SS region of pri-miR-122. (B) Agarose gel electrophoresis of PCR products generated using genomic DNA from WT Huh7 and two heterozygotic 3’SSΔ clonal cell lines (designated A and B). Expected size of PCR product was 424 bp for WT, 265 bp for 3’SSΔ. Identity of cells was confirmed by Sanger sequencing of PCR products.
